# The declining but persistent burden of lower respiratory infections from secondhand smoke in children aged under 14 years: Global trends 1990–2021 and forecasts to 2035, based on a secondary dataset analysis of Global Burden of Disease (GBD) 2021

**DOI:** 10.18332/tid/216108

**Published:** 2026-02-17

**Authors:** Fan Yang, Yiyang Zhu, Changjing Hu, Xuehong Dong, Caiping Liu, Yuxuan Li, Zifei Pan, Yue Yang, Xiaomin Jin, Qian He, Qingqing Wang, Lan Sun, Qingxia Li, Jinyang Shen

**Affiliations:** 1 Department of Pharmacy, Jiangsu Ocean University, Lianyungang, China; 2 School of Chinese Medicine, Macau University of Science and Technology, Macau, China; 3 Jiangsu Food and Pharmaceutical Science College, Huaian, China; 4 Chia Tai Tianqing Pharmaceutical Group Co., Ltd, Lianyungang, China

**Keywords:** GBD 2021, children, lower respiratory infections, secondhand smoke, prediction

## Abstract

**INTRODUCTION:**

Although global smoke-free policies have significantly reduced smoking rates, exposure to secondhand smoke (SHS) in homes and public places remains common. SHS continues to be a significant risk factor for lower respiratory infections (LRIs) in children. However, there is still a lack of systematic assessment of the spatiotemporal trends and future disease burden of LRIs attributable to SHS in children aged under 14 years.

**METHODS:**

This study is a secondary analysis of the Global Burden of Disease (GBD) 2021 data. We used joinpoint regression to analyze trends and calculate the average annual percentage change (AAPC) in the burden of LRIs attributable to SHS among children aged under 14 years, globally from 1990 to 2021. Age-standardized rates (ASRs) of mortality and DALYs were quantified at the global, regional, and national levels. Finally, a Bayesian age-period-cohort (BAPC) model was applied to forecast trends up to 2035, providing a basis for formulating targeted intervention strategies.

**RESULTS:**

In 2021, the number of deaths and disability-adjusted life years (DALYs) among children aged under 14 years worldwide due to LRIs caused by SHS decreased significantly. The ASR declined to 2.25 (95% UI: 0.73–3.86) for mortality and 199.84 (95% UI: 64.82–342.97) for DALYs per 100000 population. The forecast results indicated that by 2035, both the mortality and the DALY rates would continue to decline.

**CONCLUSIONS:**

Although the global burden had declined significantly and was expected to continue decreasing through 2035, SHS remained a significant contributor to LRIs in children.

## INTRODUCTION

Lower respiratory infections (LRIs) can be caused by pathogenic microorganisms, including viruses, *Mycoplasma, Chlamydia, and Legionella*^1^. The infection can occur directly in the lower respiratory tract, and some upper respiratory infections can also lead to LRIs by spreading downward. Among these pathogens, viruses are the primary cause^2^. LRIs mainly include infections of the bronchioles, bronchitis, and pneumonia^3^. LRIs are a leading cause of death and disability-adjusted life years (DALYs) in children under five years of age worldwide^4^. LRIs frequently occur in malnourished children and in regions with severe air pollution and low socioeconomic status^5^. However, with socioeconomic development and advances in medical care, the global age-standardized mortality rates (ASMR) from LRIs, which ranked fourth in 1990, had declined to the seventh leading cause by 2021^6^. Secondhand smoke (SHS) consists of mainstream smoke exhaled by smokers and sidestream smoke emitted from burning tobacco, both of which are diluted by ambient air^7^. More than one-third of the world’s population are passive smokers^8^. To date, over 7000 chemical substances have been identified in SHS, including at least 70 known carcinogens^9^. Approximately 600000 people die annually worldwide due to exposure to secondhand smoke, resulting in about 11 million DALYs^10^. Because children have a higher breathing rate than adults, they inhale more secondhand smoke when exposed to the same environment^11^. Bhat et al.^12^ reported that long-term exposure to SHS can reduce immunity, thereby increasing the risk of respiratory infections. Additionally, Venditto^13^ also noted that SHS can alter lung structure and impair the lungs’ defenses against infection. The gases in the smoke also inhibit the repair of damage caused by these infections^13^.

This study focuses on children aged under 14 years. The selection of this age threshold is justified on both biological and methodological grounds. First, the period from birth through early adolescence is critical for lung development and maturation of immune defenses. During this vulnerable window, chronic exposure to secondhand smoke (SHS) – a key component of household air pollution – is known to impair respiratory growth and increase susceptibility to acute lower respiratory infections, with effects that may extend into adulthood^14^. Second, from a methodological perspective, the ‘under 14 years’ definition directly aligns with the three pediatric age strata (<5, 5–9, and 10–14 years) used in the Global Burden of Disease (GBD) study. This ensures complete and comparable extraction of SHS exposure estimates and LRI burden data from the GBD database, forming a consistent basis for our trend and forecasting analyses.

## METHODS

### Data sources and estimation framework of attributable burden

This study is a secondary analysis of the Global Burden of Disease (GBD) 2021 data. The data on LRIs among children under 14 years of age attributable to SHS exposure were sourced from the GBD 2021 study. This database provides model estimates derived from exposure data and relative risks, which were accessed via the online results query tool and its companion data repository. The 2021 GBD database provides comprehensive epidemiological data from 1990 to 2021, covering 371 diseases and injuries and 88 risk factors across 204 countries^15^. It is designed to provide timely, effective, and relevant assessments of key health outcomes. To study the disease burden of LRIs caused by SHS from 1990 to 2021, data collection included variables such as gender, DALYs, mortality, and specific age, 204 countries as defined by the GBD, and 21 countries and regions with similar epidemiological characteristics. These regions and countries are categorized into five groups based on the sociodemographic index (SDI): Low (<0.46), Low-Middle (0.46–0.60), Middle (0.61–0.69), High-Middle (0.70-0.81), and High (>0.81)^16^. The SDI is a comprehensive measure of socioeconomic development, healthcare accessibility, and related factors^17^. It is estimated based on three indicators: per capita lagged distributed income, the total fertility rate among women aged ≤25 years, and the average years of education for adults aged ≥15 years^18^. A higher SDI score indicates a higher socioeconomic status.

The estimates for LRIs attributable to SHS among children aged under 14 years were extracted from the GBD 2021 study. It is crucial to clarify that ‘LRIs due to SHS’ refers not to directly observed cases but to the proportion of the total modeled burden of LRIs that is statistically attributed to SHS exposure. This attribution is performed within the GBD Comparative Risk Assessment (CRA) framework. Briefly, the CRA calculates a population attributable fraction (PAF) using two primary inputs: 1) the prevalence of exposure to SHS in the population; and 2) the relative risk (RR) of LRIs associated with SHS exposure, obtained from meta-analyses of epidemiological studies. This PAF is then applied to the total estimated burden (deaths and DALYs) of LRIs in the respective age-sex-location-year group to yield the specific burden attributable to SHS^19^. All estimates are presented with 95% uncertainty intervals (UIs), which incorporate uncertainty from multiple modeling steps and input data.

### Age-standardized rate (ASR)

Given differences in population age structures across regions and time periods, implementing age standardization is a crucial step to eliminate bias caused by these variations and ensure the comparability of results. This is achieved using a direct method based on the global age structure from the GBD study. Results are presented with a 95% uncertainty interval (UI), defined as the 2.5th and 97.5th percentiles derived from 1000 ordered draws from the posterior distribution^16,20^. The calculation formula is:


$$\text{ASR}=\frac{\Sigma_{\text{i}=1}^{\text{A}}{\text{a}}_{\text{i}}{\text{W}}_{\text{i}}}{\Sigma_{\text{i}=1}^{\text{A}}{\text{W}}_{\text{i}}}\times 100000$$

where a_i_ is the age-specific rate and W_i_ is the weight of the specific age group among the chosen population^21^.

### Average annual percentage change (AAPC)

AAPC is a summary measure of the average trend over a specified time interval, providing a single value to describe the annual rate of change across the entire study period^22^.

### Joinpoint regression analysis

To analyze trends and identify significant changes in the age-standardized rates over time, we used joinpoint regression analysis performed with the Joinpoint Regression Program (National Cancer Institute)^23^. A log-linear model was employed^24^. The analysis was divided into a maximum of 6 and a minimum of 0 joinpoints to segment the time series from 1990 to 2021. The optimal number and location of joinpoints were not pre-specified at particular milestones; instead, they were determined automatically through permutation tests^24^. Separate joinpoint analyses were conducted to characterize the distinct temporal patterns for: 1) the global population, and 2) the five sociodemographic index (SDI) groups. The AAPC for both ASMR and ASDR, along with its 95% confidence interval (CI), was then calculated as the key summary measure of the average trend over the entire 1990–2021 period. For the 204 individual countries/territories and 21 GBD regions, we report the AAPC as the summary trend metric, but did not perform or present detailed multi-segment joinpoint analyses for each entity.

### Bayesian age-period-cohort (BAPC)

This study predicted the global burden of disease from 2022 to 2035 using the Bayesian age-period-cohort (BAPC) model. The BAPC model is an age-period-cohort model within a Bayesian framework. It treats all unknown parameters as random variables, assigns appropriate prior distributions, and does not rely on explicit parameter settings. This model estimates the posterior distribution by combining prior information on unknown parameters with observed data^25^. This method addresses the inherent identifiability problem in the traditional age-period-cohort (APC) model by introducing Bayesian priors^16^. The model effectively manages age-stratified mortality data and is particularly useful for predicting future trends amid significant population changes^26^. In this study, age was stratified into three groups: <5, 5–9, and 10–14 years. A Poisson regression model was employed, with each random effect smoothed using a first-order random walk prior. The model incorporated historical observational data from 1990 to 2021 and population projection data from 2022 to 2035. Ultimately, it produced the posterior distributions of age-standardized mortality and DALY rates, along with their 95% credible intervals.

All analyses and graphical presentations in this study were performed using R (version 4.4.3)^27^. A p<0.05 was considered statistically significant.

## RESULTS

### Description of the global burden from 1990 to 2021

From 1990 to 2021, SHS has contributed to a decrease in the burden of LRIs in children worldwide (Supplementary file Table S1). The AAPC of ASMR and ASDR are -5.86 and -5.87, respectively. Specifically, the ASMR declined from 14.22 per 100000 in 1990 (95% UI: 4.8–23.62) to 2.25 per 100000 in 2021 (95% UI: 0.73–3.86). Similarly, the ASDR decreased from 1264.29 per 100000 (95% UI: 426.6–2098.92) to 199.84 per 100000 (95% UI: 64.82–342.97).

At the regional level, Oceania had the highest ASMR and ASDR in 2021 (Supplementary file Table S1). The ASMR was 15.02 per 100000 people (95% UI: 5.18–26.41), and the ASDR was 1340.49 per 100000 people (95% UI: 462.43–2356.76). While ASMR and ASDR were relatively low in Australasia: 0.05 per 100000 (95% UI: 0.01–0.08) and 4.15 per 100000 (95% UI: 1.31–7.19), respectively. The reasons for this difference are primarily related to medical and health resources, smoking rates, socioeconomic conditions, and public health policies.

### Joinpoint regression analysis of global and five SDI levels disease burden

The joinpoint regression analysis results for ASMR and ASDR (1990–2021) at the global and five SDI levels are shown in Supplementary file Figure S1. During this period, the APC for both global ASMR and ASDR demonstrated a significant downward trend (ASMR: 1990–1996 APC= -3.56; 1996–2005 APC= -5.06; 2005–2019 APC= -5.87; 2019–2021 APC= -15.70, p<0.05; ASDR:1990–1996 APC= -3.57; 1996–2005 APC= -5.05; 2005–2019 APC= -5.88; 2019–2021 APC= -15.74, p<0.05). Similarly, the same trend was observed across different SDI levels. These results highlight a notable change in mortality and DALY rates over time.

### Analysis of burdens for 204 countries and regions

**Figure 1:**
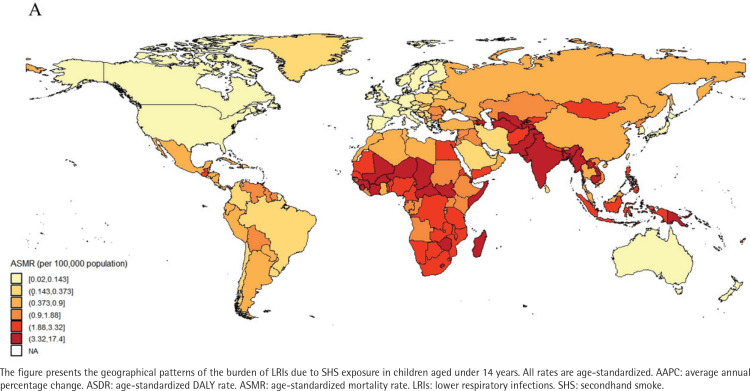
Spatial distribution of the age-standardized mortality rate (ASMR, per 100000 population) in 2021 of LRIs attributable to secondhand smoke among children aged under 14 years, 1990–2021, across 204 countries and territories

**Figure 2:**
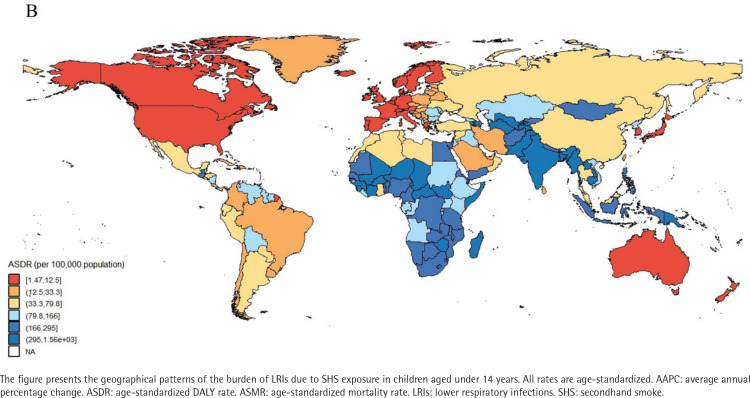
Spatial distribution of the age-standardized rates DALY rate (ASDR, per 100000 population) of LRIs attributable to secondhand smoke among children aged under 14 years, 1990–2021, across 204 countries and territories

**Figure 3:**
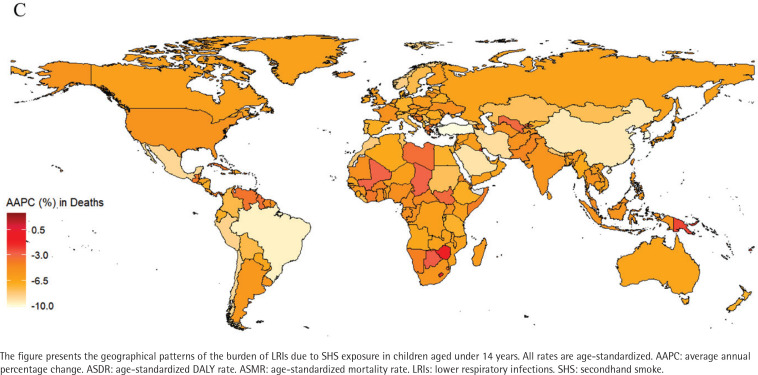
Spatial distribution of the average annual percentage change (AAPC) in ASMR from 1990 to 2021 of LRIs attributable to secondhand smoke among children aged under 14 years, 1990–2021, across 204 countries and territories

**Figure 4:**
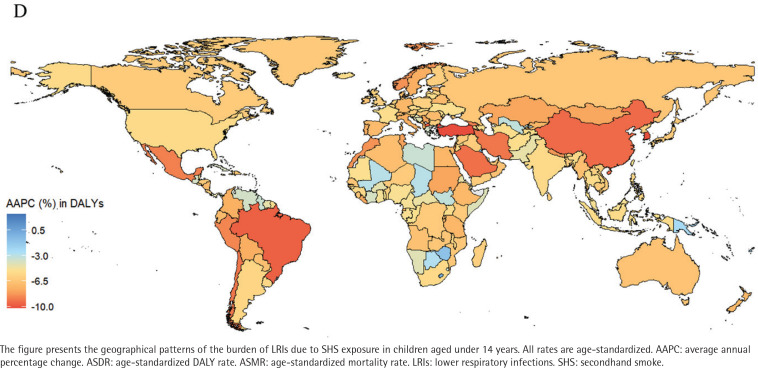
Spatial distribution of the average annual percentage change (AAPC) in ASDR from 1990 to 2021 of LRIs attributable to secondhand smoke among children aged under 14 years, 1990–2021, across 204 countries and territories

In 2021, the mortality rates from LRIs among children aged under 14 years in 204 countries and regions, ranged from 0.02 to 17.45 per 100000 people. Papua New Guinea, Tokelau, and Niue ranked among the top three in mortality rates. Papua New Guinea had 17.45 cases per 100000 (95% UI: 5.99–30.89), Tokelau had 12.64 cases per 100000 (95% UI: 4.49–21.51) and Niue had 10.29 cases per 100000 (95% UI: 3.55–17.54), while Norway had the lowest mortality rate of 0.02 per 100000 (95% UI: 0.01–0.04) (Supplementary file Table S2, and Figure 1). Similarly, data reports showed that ASDR for Papua New Guinea (1557.3; 95% UI: 535.2–2757.41), Tokelau (1113.55; 95% UI: 395.99–1893.85), and Niue (904.67; 95% UI: 312.36–1541.62) remained among the highest (Supplementary file Table S2, and Figure 2). From 1990 to 2021, the AAPC in child mortality for those aged under 14 years varied significantly among countries (Supplementary file Table S2, and Figure 3). The Republic of Korea (-10.26%; 95% CI: -10.80 – -9.72), Turkey (-10.23%; 95% CI: -10.59 – -9.86), and North Macedonia (-9.81%; 95% CI: -10.95 – -8.67) experienced the largest declines, whereas AAPC increased in Tokelau (3.13%; 95% CI: 1.31–4.97) and Niue (2.72%; 95% CI: 1.24–4.23). During the same period, the DALY rates declined most significantly in Turkey (-10.27%), the Republic of Korea (-10.18%), and North Macedonia (-9.83%) (Supplementary file Table S2, and Figure 4).

### Differences in burden at different SDI levels

From 1990 to 2021, the burden of LRIs among children aged under 14 years worldwide was significantly influenced by varying levels of the SDI. Overall, in 2021, low- and middle-income SDI regions exhibited the highest ASMR and ASDR (Supplementary file Table S1 and Figure S2), with an ASMR of 3.38 per 100000 people (95% UI: 1.1–5.85) and an ASDR of 301.03 per 100000 people (95% UI: 98.12 – 520.67). Inter-group comparisons revealed that the UI burden in high SDI regions (0.09; 95% UI: 0.03–0.15) did not overlap with that in low SDI regions (3.33; 95% UI: 1.04–5.89) or low-to-mid SDI regions (3.38; 95% UI: 1.10–5.85), indicating highly statistically significant differences^28^. Looking at different genders (Supplementary file Table S1 and Figure S2A), the ASMR for males in low-middle SDI regions decreased from 19.21 cases per 100000 in 1990 (95% UI: 6.39–32.19) to 3.44 cases per 100000 in 2021 (95% UI: 1.1–5.94); the ASDR decreased from 1710.61 cases per 100000 in 1990 (95% UI: 569.55–2865.3) to 306.02 cases per 100000 in 2021 (95% UI: 98.39–529.13). The female ASMR dropped from 20.51 per 100000 (95% UI: 6.71–35.86) to 3.33 per 100000 (95% UI: 1.05–5.73), while the ASDR declined from 1822.57 per 100000 (95% UI: 596.13–3186.43) to 295.72 per 100000 (95% UI: 93.44–509.43) in 2021. Within the same SDI group, the estimated disease burden for males and females was very similar, with their uncertainty intervals nearly completely overlapping, indicating no statistically significant gender differences. It was clearly observed from Supplementary file Table S1 and Figure S2B that the AAPC in ASMR and ASDR tended to be consistent, with the greatest decrease in AAPC occurring in the high-middle SDI (-8.27%).

### The relationship between 21 GBD regions and SDI

Among the 21 GBD regions, the disease burden in children aged under 14 years was significantly and linearly related to the SDI (Supplementary file Figures S3A and S3C). The x-axis represents the sociodemographic index (SDI), ranging from 0 to 1, with higher values indicating greater levels of development. The y-axis displays the age-standardized rate. Spearman correlation analysis showed a significant negative correlation between SDI and ASMR (ρ= -0.8390, p<0.001). However, there was no significant correlation between the AAPC of ASMR from 1990 to 2021 and SDI (ρ= -0.275, p=0.2155). Similarly, a significant negative correlation was found between SDI and the ASDR (ρ= -0.8385, p<0.001), while the AAPC of ASDR showed no significant correlation with SDI (ρ= -0.2847, p=0.1991) (Supplementary file Figures S3B and S3D). These findings indicate that the rate of improvement in disease burden across regions with different SDI levels globally is not solely determined by their SDI level. Among these regions, the disease burden in Tropical Latin America, East Asia, and the High-income Asia-Pacific experienced the most significant declines (Supplementary file Table S1 and Figure S3). The ASMR in Tropical Latin America fell from 8.28 (95% 2.72–13.54) in 1990 to 0.38 (95% UI: 0.11– 0.68) in 2021. In East Asia, it decreased from 17.46 (95% UI: 6.06–29.23) to 0.85 (95% UI: 0.29–1.41), and in the high-income Asia-Pacific, it declined from 0.77 (95% UI: 0.27–1.29) to 0.06 (95% UI: 0.02–0.10). The disease burden in other regions has decreased slightly Supplementary file Table S1 and Figure S3A). Regarding the ASDR, the largest declines are also observed in these three regions (Supplementary file Table S1 and Figure S3B).

### BAPC’s prediction of ASMR and ASDR

To understand the global trends in children aged under 14 years after 2021 regarding LRIs due to SHS, ASMR, and ASDR from 2021 to 2035, the Bayesian age-period-cohort (BAPC) model was used. The solid line denotes the model-based estimates for the historical period (1990–2021). The dashed line to the right corresponds to the projected values from 2022 onward. The shaded area represents the 95% uncertainty interval for the age-standardized rate during the forecast period. The forecast indicates that the overall disease burden will continue to decline over time. The ASMR is projected to decrease from 2.25 per 100000 in 2021 to 0.41 per 100000 in 2035. Similarly, the ASDR is expected to decline from 199.84 per 100000 in 2021 to 38.56 per 100000 in 2035 (Supplementary file Table S3 and Figure S4).

## DISCUSSION

This study comprehensively assessed the long-term trends in mortality and disability-adjusted life years (DALYs) due to lower respiratory infections attributable to secondhand smoke exposure among children aged under 14 years from 1990 to 2021. By integrating GBD data and incorporating predictive models, it aims to provide a reference for future disease burden assessments and to inform priority-setting in public health resource allocation. The results indicated that the ASMR in this age group declined significantly worldwide. However, the absolute number of deaths remained substantial. This trend may be associated with the interaction between population growth and tobacco exposure^29^. Although the overall global trend was improving, the burden of LRIs varied considerably across different countries and regions. Australia and New Zealand exhibit relatively low mortality rates, and their successful experiences suggest a possible association with tobacco control legislation. Australia was the first country to enact legislation mandating plain packaging for tobacco products^30^. New Zealand had implemented the Smoke-free Environment Amendment Act (SEAA), which banned smoking in nearly all public places, extending existing restrictions in offices and retail environments^31^. However, some island countries in Oceania faced significant challenges in health services due to geographical isolation and limited land area^32^. Extensive interference and sabotage by the tobacco industry had undermined the effectiveness of tobacco control measures and public health policies in certain countries^33^. Taking Papua New Guinea as an example, the country was densely populated and had a high smoking rate^34^. Among these smokers, 87% reside in rural communities where access to medical services is limited. Hospitals were primarily located in urban areas, and for most rural residents, utilizing these medical facilities involved high costs^35^. In addition, deficiencies in legislative capacity impede policy implementation. In response to the disparities outlined above, the following recommendations are proposed for regions with a high disease burden: increasing the consumption tax on tobacco, which was considered^34^ an important and effective step to reduce global tobacco use; learning from the experiences of Australia and New Zealand by formulating and enforcing stricter tobacco control laws and restrictions on tobacco advertising and promotions; and increasing investment in areas with limited healthcare resources is expected to enhance the prevention, diagnosis, and treatment of respiratory infections in children, thereby potentially reducing the associated disease burden.

### Limitations

Despite the extensive coverage of GBD databases and the detailed data provided, this study has several limitations that should be considered when interpreting the results. First, and most pertinent to the core estimates of this study, the burden of LRIs attributable to SHS is subject to substantial uncertainty inherent in the modeling process. As detailed in the Methods, these estimates are derived from the GBD Comparative Risk Assessment (CRA) framework. Their uncertainty originates from two primary, interconnected layers: 1) uncertainty in the underlying estimates of the total incidence, prevalence, and mortality of LRIs in children, which is heavily dependent on the availability, quality, and completeness of source data (e.g. potential under-reporting or diagnostic variability, particularly in resource-limited settings); and 2) uncertainty in the estimates of SHS exposure distribution and, critically, in the relative risk (RR) linking SHS exposure to LRIs. The RR estimates are derived from meta-analyses of observational studies, meaning any residual confounding, measurement error, or bias in these source studies is propagated into the calculation of the population attributable fraction and, consequently, the final burden estimates. While the 95% uncertainty intervals (UIs) reported throughout our results quantitatively reflect this combined uncertainty, the absolute magnitude of the SHS-attributable burden should be interpreted with appropriate caution. Second, building on the above, the GBD data themselves are comprehensive model-based syntheses. Variations in data collection standards, reporting practices, and healthcare access across the 204 countries and territories can lead to discrepancies between modeled estimates and true local disease burden. For instance, significant gaps have been noted between GBD estimates and national surveillance data for certain conditions, such as dengue fever in China^36^. This underscores that while the GBD provides invaluable globally comparable metrics, local validation remains important. Third, our analysis, being based on population-level estimates, cannot account for all individual-level confounding factors that may influence both SHS exposure and LRI risk, such as detailed household socioeconomic status, parental education, indoor air pollution from other sources, or nutritional status. The use of the sociodemographic index (SDI) as an ecological-level covariate partly addresses this but cannot eliminate individual-level confounding. Fourth, there is an inherent time lag in the GBD data, with the current analysis extending only to 2021. Consequently, our findings cannot reflect the impact of very recent policy changes, public health campaigns, or major events that occurred after this cutoff. Fifth, our predictions for the period up to 2035, generated using the BAPC model, are based on past trends and demographic projections. While the model accounts for age, period, and cohort effects, its accuracy may be reduced by unforeseen future events, disruptions in health systems, or non-linear changes in tobacco control policies and SHS exposure. Finally, this is an exploratory ecological study using large-scale public data. We did not employ formal statistical correction methods, which increases the risk of Type I error when interpreting specific subgroup trends. However, the primary global and sustained downward trends reported are robust, and the identified patterns, such as the higher persistent burden in low-SDI regions, offer critical preliminary evidence and a conceptual framework for prioritizing public health interventions. Future confirmatory studies and local investigations should apply more granular data and rigorous causal inference methods to validate and build upon these associations.

## CONCLUSIONS

Through an analysis of the burden of SHS attributable to LRIs in children aged under 14 years, worldwide from 1990 to 2021, along with forecasts through 2035, the study found that mortality rates were expected to continue declining. The research reveals significant differences across regions and genders, with economically underdeveloped areas facing particular challenges. These findings provide an important reference for policymakers to develop targeted solutions.

## Supplementary Material



## Data Availability

The data supporting this research are available from the following sources: Model estimates derived from exposure data and relative risks, accessed via the online results query tool (https://vizhub.healthdata.org/gbd-results/) and its companion data repository (https://ghdx.healthdata.org/).
