# Supplementary material for: The declining but persistent burden of lower respiratory infections from secondhand smoke in children aged under 14 years: Global trends 1990–2021 and forecasts to 2035, based on a secondary dataset analysis of Global Burden of Disease (GBD) 2021
Source: Tob Induc Dis. 2026 Feb 17;24:10.18332/tid/216108. doi: 10.18332/tid/216108 (PMC12911320; doi:10.18332/tid/216108)
Supplement: Supplementary file 1 [file TID-24-21-s1.pdf]

Supplementary Figure S1. Average percentage change (APC) in age-standardized mortality rate (ASMR) and age-standardized disability-adjusted life year rate (ASDR) of lower respiratory infections (LRIs) attributable to secondhand smoke (SHS) in children under 14, 1990–2021.

(A) Global Trends in ASMR. (B) Global Trends in ASDR. (C) High SDI Trends in ASMR. (D) High SDI Trends in ASDR. (E) High-middle SDI Trends in ASMR. (F) High-middle SDI Trends in ASDR. (G) Middle SDI Trends in ASMR. (H) Middle SDI Trends in ASDR. (I) Low-middle SDI Trends in ASMR. (J) Low-middle SDI Trends in ASDR. (K) Low SDI Trends in ASMR. (L) Low SDI Trends in ASDR.

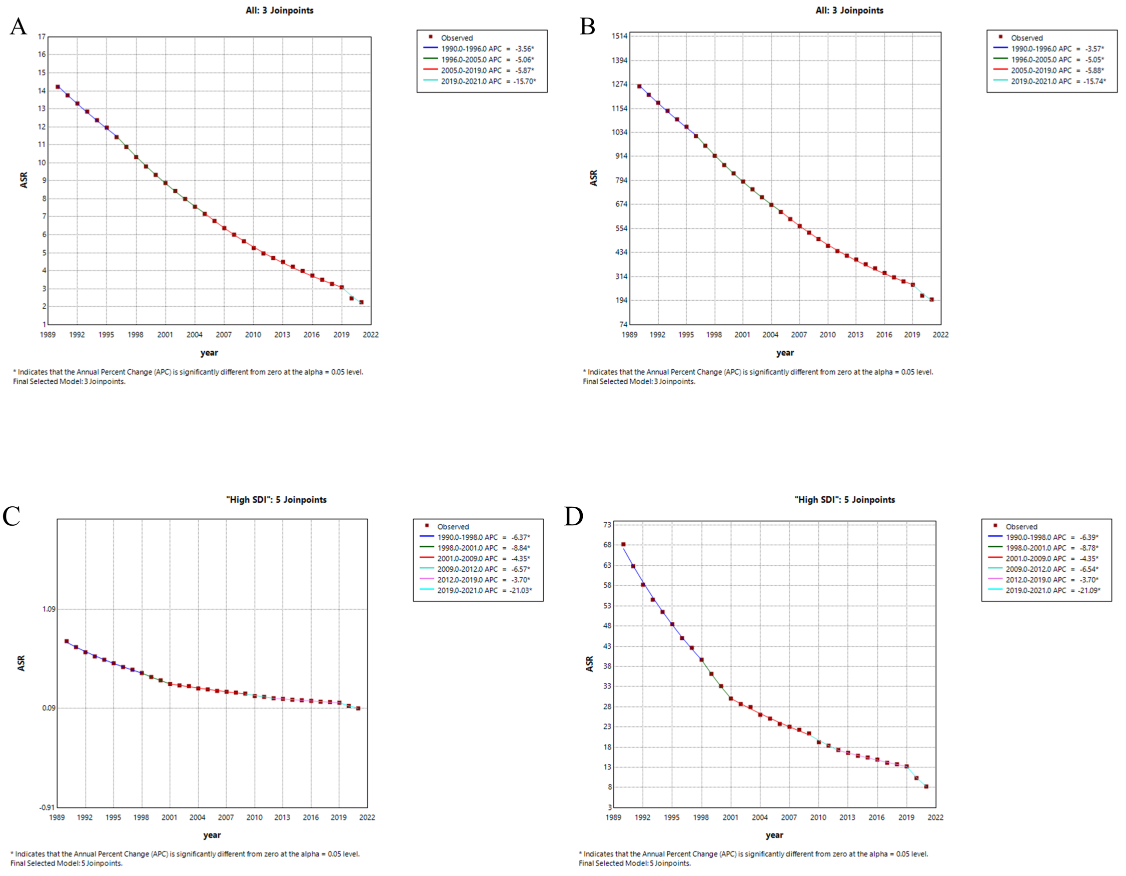

E F

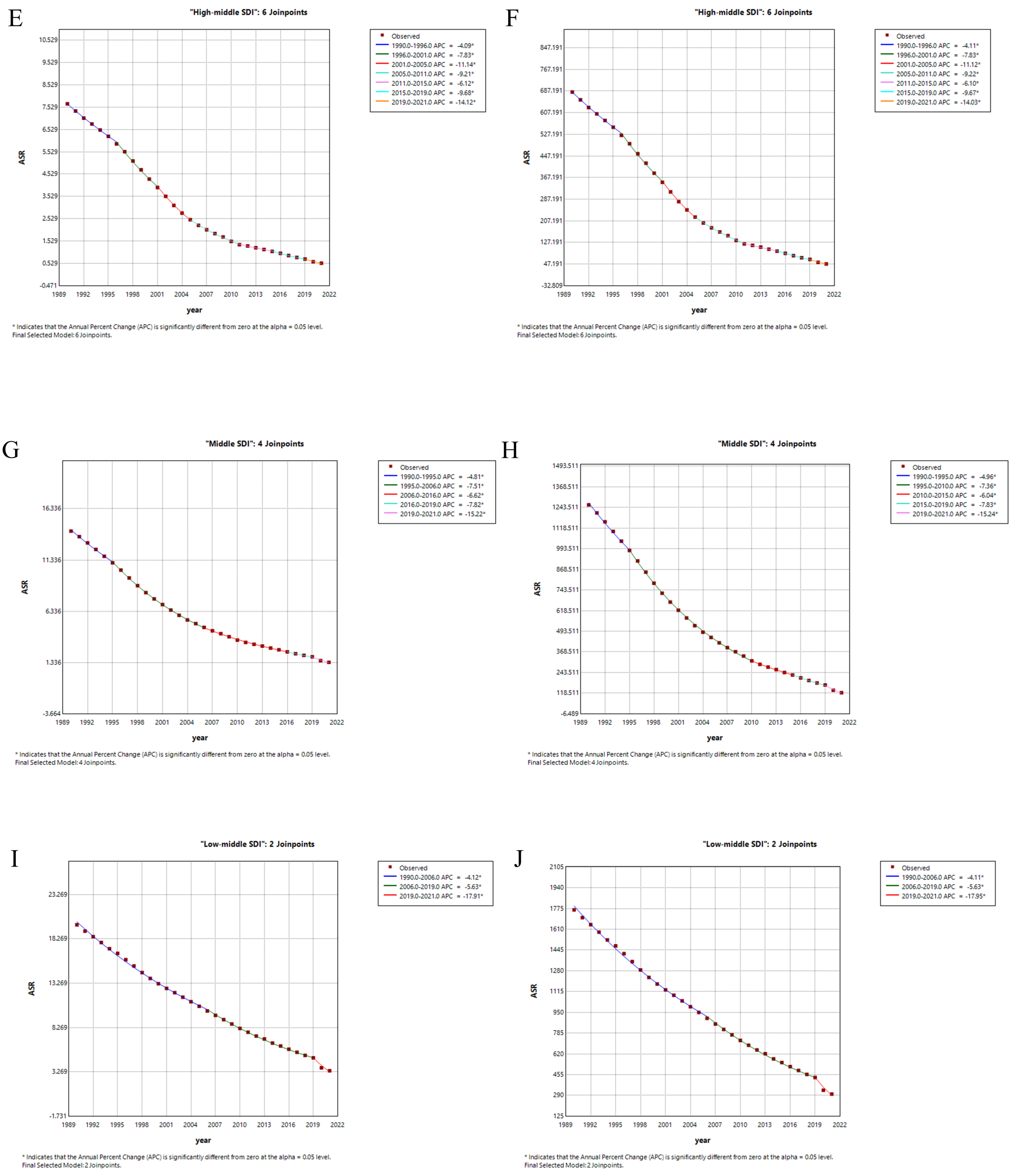
"High-middle SDI": 6 Joinpoints
Observed
1990.0-1996.0 APC = -4.09*
1996.0-2001.0 APC = -7.83*
2001.0-2005.0 APC = -11.14*
2005.0-2011.0 APC = -9.21*
2011.0-2015.0 APC = -6.12*
2015.0-2019.0 APC = -9.68*
2019.0-2021.0 APC = -14.12*
ASR
year
* Indicates that the Annual Percent Change (APC) is significantly different from zero at the alpha = 0.05 level.
Final Selected Model: 6 Joinpoints.
"High-middle SDI": 6 Joinpoints
Observed
1990.0-1996.0 APC = -4.11*
1996.0-2001.0 APC = -7.83*
2001.0-2005.0 APC = -11.12*
2005.0-2011.0 APC = -9.22*
2011.0-2015.0 APC = -6.10*
2015.0-2019.0 APC = -9.67*
2019.0-2021.0 APC = -14.03*
ASR
year
* Indicates that the Annual Percent Change (APC) is significantly different from zero at the alpha = 0.05 level.
Final Selected Model: 6 Joinpoints.
G
"Middle SDI": 4 Joinpoints
Observed
1990.0-1995.0 APC = -4.81*
1995.0-2006.0 APC = -7.51*
2006.0-2016.0 APC = -6.62*
2016.0-2019.0 APC = -7.82*
2019.0-2021.0 APC = -15.22*
ASR
year
* Indicates that the Annual Percent Change (APC) is significantly different from zero at the alpha = 0.05 level.
Final Selected Model: 4 Joinpoints.
H
"Middle SDI": 4 Joinpoints
Observed
1990.0-1995.0 APC = -4.96*
1995.0-2010.0 APC = -7.36*
2010.0-2015.0 APC = -6.04*
2015.0-2019.0 APC = -7.83*
2019.0-2021.0 APC = -15.24*
ASR
year
* Indicates that the Annual Percent Change (APC) is significantly different from zero at the alpha = 0.05 level.
Final Selected Model: 4 Joinpoints.
I
"Low-middle SDI": 2 Joinpoints
Observed
1990.0-2006.0 APC = -4.12*
2006.0-2019.0 APC = -5.63*
2019.0-2021.0 APC = -17.91*
ASR
year
* Indicates that the Annual Percent Change (APC) is significantly different from zero at the alpha = 0.05 level.
Final Selected Model: 2 Joinpoints.
J
"Low-middle SDI": 2 Joinpoints
Observed
1990.0-2006.0 APC = -4.11*
2006.0-2019.0 APC = -5.63*
2019.0-2021.0 APC = -17.95*
ASR
year
* Indicates that the Annual Percent Change (APC) is significantly different from zero at the alpha = 0.05 level.
Final Selected Model: 2 Joinpoints.

K

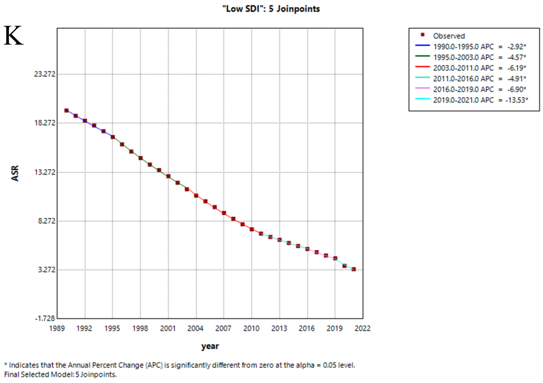

L

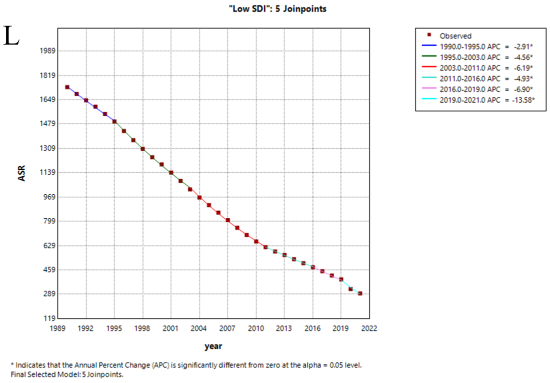

Note: The asterisk (*) indicates p-value < 0.05, denoting a statistically significant APC (i.e., significantly different from zero). The SDI quintiles are: high, high-middle, middle, low-middle, and low. ASMR, age-standardized mortality rate; ASDR, age-standardized disability-adjusted life year rate.

Supplementary Figure S2. Temporal trends and average annual percentage change (AAPC) in the ASMR and ASDR of LRIs attributable to SHS, by gender and Socio-demographic Index (SDI) region, 1990–2021.

(A) Trends of ASMR and ASDR in Different SDI Levels. (B) Trends of AAPC in Different SDI Levels.

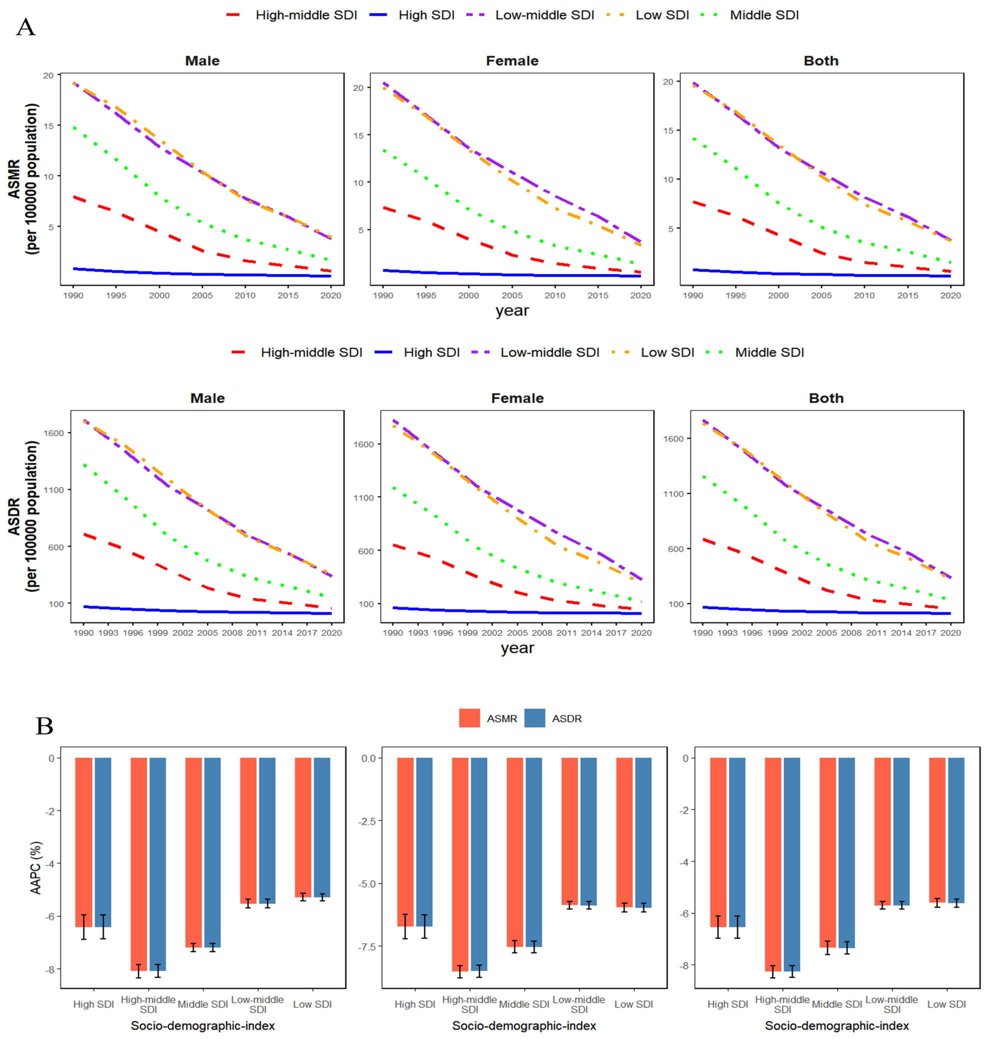

Note: The SDI quintiles are: high, high-middle, middle, low-middle, and low. ASMR, age-standardized mortality rate; ASDR, age-standardized disability-adjusted life year rate; AAPC, average annual percentage change; LRI, lower respiratory infection; SHS, secondhand smoke; Error bars, 95% CI for the AAPC.

Supplementary Figure S3. Regional correlations between the SDI and the burden of LRIs attributable to SHS in children under 14, 1990–2021.

(A) Correlation between ASMR and SDI. (B) Correlation between ASDR and SDI. (C) Correlation between the AAPC of ASMR and SDI. (D) Correlation between the AAPC of ASDR and SDI.

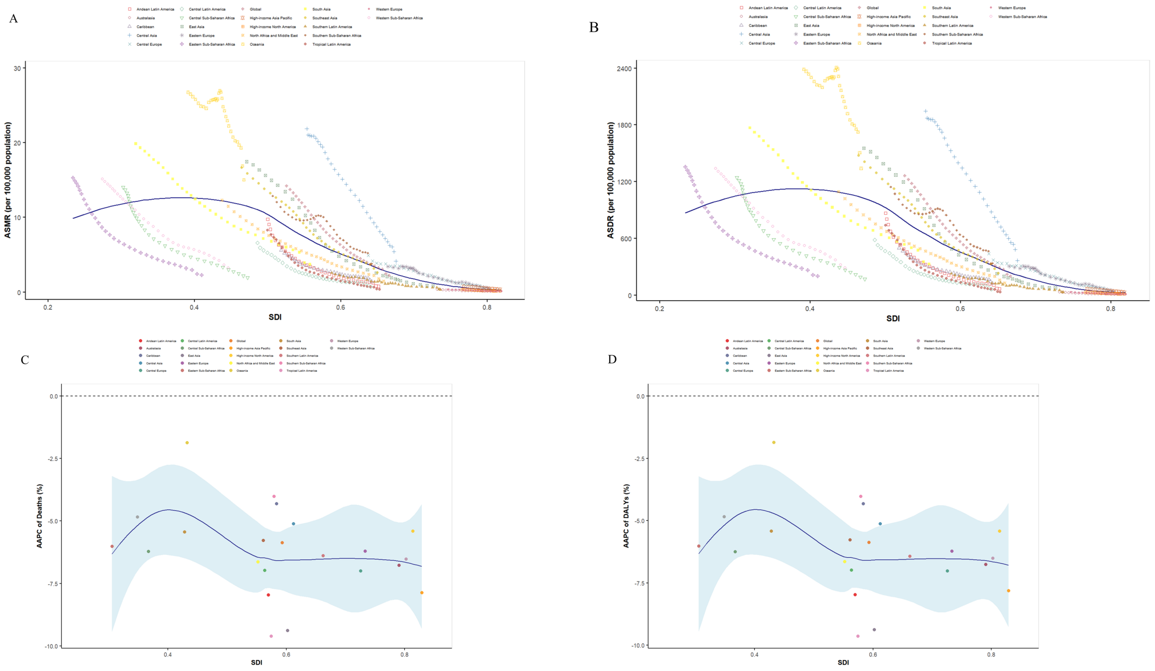

Note: SDI, Socio-demographic Index; ASMR, age-standardized mortality rate; ASDR, age-standardized disability-adjusted life year rate; AAPC, average annual percentage change; LRI, lower respiratory infection; SHS, secondhand smoke; Fitted curve, the nonlinear relationship between SDI and AAPC; The shaded area , the 95% confidence interval (CI) of the fitted curve.

Supplementary Figure S4. Projected global trends in the ASMR and ASDR of LRIs attributable to SHS in children under 14, 2022–2035, modeled using the Bayesian age-period-cohort framework. (A) Age-standardized mortality rate (ASMR). (B) Age-standardized disability-adjusted life year rate (ASDR).

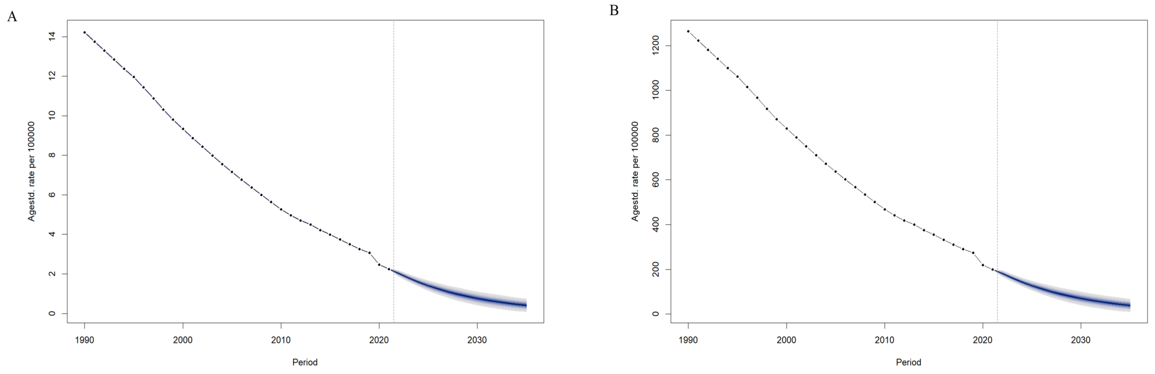

Note: The shaded blue area represents the 95% confidence interval. LRI, lower respiratory infection; SHS, secondhand smoke.

Supplementary Table S1 Mortality, DALYs counts, ASRs, and AAPC (%) under 14 years old across global, SDI, and GBD regions from 1990 to 2021.

	1990		2021		
location	Counts (95% UI)	ASR per 100,000 (95% UI)	Counts (95% UI)	ASR per 100,000 (95% UI)	AAPC from 1990 to 2021 (95% CI)
Deaths					
Global	251627.08 (85033.91-417711.65)	14.22 (4.8-23.62)	42656.89 (13830.08-73323.88)	2.25 (0.73-3.86)	-5.86 (-5.99 - -5.74)
High SDI	1373.67 (474.68-2255.61)	0.77 (0.27-1.27)	149.34 (50.33-245.73)	0.09 (0.03-0.15)	-6.55 (-6.98 - -6.11)
Male	770.13 (268.77-1266.36)	0.84 (0.29-1.38)	87.4 (29.33-144.93)	0.11 (0.04-0.18)	-6.42 (-6.88 - -5.96)
Female	603.54 (205.23-985.09)	0.7 (0.24-1.13)	61.95 (20.91-101.21)	0.08 (0.03-0.13)	-6.72 (-7.21 - -6.24)
High-middle SDI	20413.42 (7117.42-33830.9)	7.68 (2.66-12.66)	1104.89 (378.41-1790.06)	0.53 (0.18-0.87)	-8.27 (-8.50 - -8.03)
Male	11001.31 (3846.54-18236.72)	7.96 (2.77-13.21)	640.12 (218.03-1043.69)	0.59 (0.2-0.97)	-8.09 (-8.34 - -7.84)
Female	9412.11 (3283.69-15632.21)	7.37 (2.56-12.21)	464.77 (158.8-759.01)	0.47 (0.16-0.77)	-8.52 (-8.76 - -8.28)
Middle SDI	81149.68 (28247.41-134019.76)	14.16 (4.93-23.42)	7003.23 (2318.73-11794.65)	1.36 (0.45-2.3)	-7.34 (-7.61 - -7.08)
Male	44108.24 (15242.51-73209.88)	14.86 (5.14-24.66)	4003.58 (1343.4-6723.23)	1.5 (0.5-2.52)	-7.19 (-7.35 - -7.04)
Female	37041.44 (12578.29-62268.27)	13.41 (4.58-22.49)	2999.65 (975.34-5034.6)	1.22 (0.4-2.05)	-7.53 (-7.77 - -7.29)
Low-middle SDI	98095.92 (32984.83-163654.12)	19.84 (6.68-33.12)	18624.76 (6055.13-31880.05)	3.38 (1.1-5.85)	-5.70 (-5.86 - -5.55)
Male	48784.61 (16257.99-81523.02)	19.21 (6.39-32.19)	9748.59 (3126.5-16783.86)	3.44 (1.1-5.94)	-5.53 (-5.69 - -5.37)
Female	49311.31 (16186.57-85902.33)	20.51 (6.71-35.86)	8876.16 (2823.62-15161.36)	3.33 (1.05-5.73)	-5.88 (-6.02 - -5.73)
Low SDI	50440.48 (16143.47-87576.49)	19.59 (6.27-33.99)	15718.94 (4900.84-27661.67)	3.33 (1.04-5.89)	-5.61 (-5.77 - -5.45)
Male	25179.05 (7965.69-44294.78)	19.19 (6.06-33.71)	8715.57 (2646.18-15358.91)	3.62 (1.1-6.38)	-5.29 (-5.43 - -5.15)
Female	25261.43 (7991.21-44082.68)	20 (6.28-34.81)	7003.37 (2113.81-12236.5)	3.04 (0.92-5.36)	-5.97 (-6.14 - -5.80)
Andean Latin America	1470.97 (480.92-2559.06)	9.76 (3.19-17)	135.18 (39.68-247.45)	0.76 (0.23-1.41)	-7.95 (-8.44 - -7.46)
Australasia	17.54 (6.12-28.61)	0.4 (0.14-0.65)	2.47 (0.78-4.21)	0.05 (0.01-0.08)	-6.77 (-8.01 - -5.51)
Caribbean	660.69 (203.58-1200.93)	5.61 (1.73-10.21)	155.76 (47.82-285.45)	1.4 (0.43-2.61)	-4.31 (-4.52 - -4.11)
Central Asia	5922.93 (2016.22-9901.44)	21.86 (7.44-36.52)	1172.66 (388.7-1969.91)	4.12 (1.36-6.92)	-5.11 (-5.27 - -4.95)
Central Europe	1293.51 (456.11-2104.98)	4.91 (1.72-7.99)	81.13 (27.51-132.35)	0.5 (0.17-0.81)	-7.00 (-7.64 - -6.36)
Central Latin America	4319.05 (1447.24-7170.36)	6.58 (2.2-10.92)	410.6 (128.54-741.67)	0.7 (0.22-1.28)	-6.97 (-7.39 - -6.54)
Central Sub-Saharan Africa	4119.68 (1139.82-7528.97)	14.02 (3.91-25.91)	1153.19 (316.1-2145.25)	1.93 (0.54-3.58)	-6.22 (-6.32 - -6.12)
East Asia	57708.5 (20078.81-96765.4)	17.46 (6.06-29.23)	2011.5 (693.66-3322.06)	0.85 (0.29-1.41)	-9.38 (-9.86 - -8.89)
Eastern Europe	1689.38 (601.3-2715.89)	3.42 (1.21-5.5)	139.2 (48.58-232.66)	0.46 (0.16-0.76)	-6.20 (-6.96 - -5.44)
Eastern Sub-Saharan Africa	15645.05 (4898.27-27285.83)	15.33 (4.79-26.76)	4170.1 (1261.94-7485.76)	2.3 (0.69-4.14)	-6.01 (-6.23 - -5.78)
High-income Asia Pacific	241.32 (83.26-395.56)	0.77 (0.27-1.29)	12.71 (4.3-21.1)	0.06 (0.02-0.1)	-7.86 (-8.43 - -7.29)
High-income North America	218.23 (73.99-359.85)	0.35 (0.12-0.58)	37.52 (12.08-64.53)	0.06 (0.02-0.11)	-5.40 (-6.29 - -4.51)
North Africa and Middle East	17966.51 (5991.59-30701.21)	12.31 (4.09-21.07)	2569.12 (836.63-4363.76)	1.46 (0.47-2.51)	-6.63 (-6.78 - -6.47)
Oceania	766.54 (254.93-1274.19)	26.77 (8.9-44.65)	827.87 (288.5-1445.91)	15.02 (5.18-26.41)	-1.86 (-2.13 - -1.59)
South Asia	88995.89 (29562.24-149806.41)	19.87 (6.59-33.71)	16910.7 (5362.66-29548.99)	3.7 (1.16-6.51)	-5.43 (-5.78 - -5.07)
Southeast Asia	27797.16 (9600.55-46005.19)	16.66 (5.73-27.62)	4280.79 (1449.08-7230.89)	2.64 (0.89-4.45)	-5.77 (-5.86 - -5.67)
Southern Latin America	375.48 (124.72-609.24)	2.55 (0.85-4.14)	43.57 (14.1-72.83)	0.34 (0.11-0.57)	-6.38 (-7.34 - -5.41)
Southern Sub-Saharan Africa	2740.68 (891.66-4614.47)	12.87 (4.19-21.75)	875.7 (283.48-1523.47)	3.79 (1.22-6.58)	-4.01 (-4.31 - -3.71)
Tropical Latin America	4067.58 (1336.32-6636.08)	8.28 (2.72-13.54)	186.98 (57.8-327.93)	0.38 (0.11-0.68)	-9.61 (-10.16 - -9.06)
Western Europe	250.76 (87.24-405.98)	0.38 (0.13-0.61)	29.85 (9.97-49.84)	0.05 (0.02-0.08)	-6.52 (-7.51 - -5.52)
Western Sub-Saharan Africa	15359.64 (4580.15-26721.74)	15.16 (4.52-26.41)	7450.31 (2257.49-13395.69)	3.28 (1-5.91)	-4.84 (-5.00 - -4.68)
DALYs					
Global	22369909.56 (7561735.75-37116508.55)	1264.29 (426.6-2098.92)	3785173.2 (1227342.44-6506457.53)	199.84 (64.82-342.97)	-5.87 (-6.00 - -5.74)
High SDI	121511.19 (42032.19-199530.57)	68.25 (23.61-112.35)	13178.21 (4444.52-21617.59)	8.25 (2.77-13.59)	-6.55 (-6.98 - -6.12)
Male	68177.31 (23803.1-112079.48)	74.65 (25.99-122.31)	7725.27 (2592.56-12816.52)	9.46 (3.14-15.72)	-6.42 (-6.87 - -5.96)
Female	53333.88 (18160.66-87089.33)	61.49 (20.94-100.28)	5452.94 (1847.89-8923.4)	6.98 (2.35-11.56)	-6.73 (-7.20 - -6.25)
High-middle SDI	1816619.48 (633664.26-3008884.3)	683.27 (236.71-1126.63)	98430.42 (33707.76-159140.63)	47.71 (16.46-77.55)	-8.26 (-8.49 - -8.03)
Male	979276.7 (342583.29-1622510.5)	708.75 (246.48-1175.81)	56954.92 (19383.43-92815.76)	52.67 (18.24-86.56)	-8.08 (-8.33 - -7.83)
Female	837342.78 (292127.12-1390999.19)	655.75 (227.95-1086.99)	41475.5 (14208.87-67853.72)	42.24 (14.48-69.25)	-8.51 (-8.75 - -8.26)
Middle SDI	7215551.16 (2513514.4-11918906.51)	1258.9 (439.03-2082.52)	620551.53 (205488.48-1044672.51)	120.9 (39.93-203.88)	-7.36 (-7.59 - -7.12)
Male	3924614.49 (1356679.19-6514119.84)	1322.02 (457.69-2194.16)	354873.89 (119098.7-596608.64)	132.78 (44.48-223.8)	-7.20 (-7.35 - -7.04)
Female	3290936.67 (1118505.26-5531520.4)	1191.08 (407.18-1997.56)	265677.64 (86389.77-445600.6)	107.98 (35.1-181.63)	-7.53 (-7.77 - -7.30)
Low-middle SDI	8727625.08 (2935010.69-14562051.72)	1765.05 (594.11-2945.8)	1656192.67 (538348.4-2837588.34)	301.03 (98.12-520.67)	-5.70 (-5.85 - -5.55)
Male	4345461.13 (1448212.38-7259444.52)	1710.61 (569.55-2865.3)	867564.06 (278367.14-1495090.09)	306.02 (98.39-529.13)	-5.53 (-5.69 - -5.37)
Female	4382163.95 (1438801.92-7636435.84)	1822.57 (596.13-3186.43)	788628.61 (250607.78-1346530.43)	295.72 (93.44-509.43)	-5.88 (-6.04 - -5.73)
Low SDI	4474911.36 (1432807.59-7763072.73)	1736.93 (556.18-3013.85)	1391858.78 (434285.06-2449929.74)	295.08 (92.02-520.98)	-5.62 (-5.77 - -5.46)
Male	2235439.86 (707628.3-3933597.11)	1702.96 (537.78-2991.96)	772229.22 (234776.75-1360663.34)	320.43 (97.22-565.36)	-5.29 (-5.43 - -5.15)
Female	2239471.5 (708608.19-3905410.03)	1772.22 (556.48-3082.72)	619629.56 (187067.22-1083730.28)	268.59 (81.3-473.97)	-5.98 (-6.15 - -5.80)
Andean Latin America	130699.44 (42724.06-227341.08)	867.12 (283.02-1510.27)	11919.88 (3491.11-21818.91)	67.42 (19.88-124.1)	-7.97 (-8.46 - -7.48)
Australasia	1561.49 (544.83-2550.4)	35.29 (12.38-57.75)	219.81 (69.11-376.65)	4.15 (1.31-7.19)	-6.76 (-8.00 - -5.51)
Caribbean	58789.5 (18118.48-106925.14)	499.06 (153.93-908.55)	13841.88 (4244-25386.82)	124.82 (38.16-231.56)	-4.32 (-4.52 - -4.11)
Central Asia	527332.11 (179526.79-881432.05)	1945.38 (662.03-3249.89)	104052.09 (34476.36-174728.45)	365.42 (120.27-613.54)	-5.12 (-5.28 - -4.96)
Central Europe	115008.83 (40555.44-187134)	436.71 (153.54-710.75)	7162.64 (2426.02-11672.78)	43.86 (14.79-71.78)	-7.02 (-7.65 - -6.38)
Central Latin America	383993.39 (128674.29-637568.03)	585.03 (195.8-970.98)	36370.62 (11378.4-65713.68)	62.52 (19.46-113.66)	-6.98 (-7.40 - -6.55)
Central Sub-Saharan Africa	365554.92 (101204.41-668648.93)	1243.2 (346.51-2296.88)	101616.74 (27910.29-189354.63)	169.59 (47.15-314.89)	-6.24 (-6.34 - -6.14)
East Asia	5134721.1 (1787172.62-8609467.69)	1553.52 (539.67-2601.28)	179206.91 (61821.04-295833.58)	76.26 (26.3-125.98)	-9.37 (-9.84 - -8.89)
[illegible]	150727.61 [illegible]	[illegible]	12316.22 [illegible]	[illegible]	[illegible]

Eastern Europe	[illegible] (53670.64-242194.02)	305.02 (108.46-490.28)	[illegible] (4300.39-20624.29)	40.76 (14.07-67.89)	-6.22 (-6.98 - -5.45)
Eastern Sub-Saharan Africa	1386682.26 (434491.67-2417786.78)	1357.25 (424.7-2369.81)	367741.46 (111358.24-660562.39)	202.5 (61.31-365.35)	-6.02 (-6.24 - -5.80)
High-income Asia Pacific	21123.68 (7313.54-34589.64)	68.13 (23.65-113.19)	1127.61 (381.44-1873.17)	5.57 (1.84-9.28)	-7.81 (-8.36 - -7.25)
High-income North America	19346.78 (6567.74-31890.12)	31.26 (10.63-51.66)	3292.51 (1060-5656.9)	5.41 (1.71-9.33)	-5.42 (-6.36 - -4.46)
North Africa and Middle East	1596885.81 (532176.29-2732180.86)	1093.92 (362.99-1872.14)	226986.03 (73856.27-385801.88)	128.86 (41.75-222.08)	-6.64 (-6.80 - -6.49)
Oceania	68435.11 (22754.29-113744.66)	2389.73 (793.96-3983.74)	73893.98 (25726.05-129080.48)	1340.49 (462.43-2356.76)	-1.86 (-2.13 - -1.59)
South Asia	7923193.68 (2632064.93-13338033.58)	1768.31 (587.24-2999.27)	1506867.47 (477363.97-2636576.57)	329.81 (103.46-580.29)	-5.42 (-5.77 - -5.07)
Southeast Asia	2465061.24 (851796.96-4079368.16)	1477.32 (508.79-2448.63)	379377.24 (128411.39-640805.73)	233.89 (78.95-394.8)	-5.77 (-5.86 - -5.67)
Southern Latin America	33484.29 (11130.47-54343.17)	227.46 (75.53-369.32)	3817.58 (1234.53-6391.78)	29.59 (9.54-50.08)	-6.43 (-7.39 - -5.46)
Southern Sub-Saharan Africa	243538.93 (79285.36-410220.39)	1143.41 (372.1-1932.71)	77425.86 (25067.15-134711.17)	335.05 (108.21-581.52)	-4.02 (-4.33 - -3.72)
Tropical Latin America	362362.8 (119021.08-591395.53)	738.29 (242.29-1205.99)	16512.21 (5093.8-28945.16)	33.41 (10.14-59.68)	-9.64 (-10.18 - -9.09)
Western Europe	22117.25 (7700.37-35801.78)	33.2 (11.55-53.67)	2632.7 (878.46-4385.61)	4.17 (1.39-7)	-6.51 (-7.49 - -5.52)
Western Sub-Saharan Africa	1359289.34 (405693.09-2364927.62)	1340.7 (399.83-2334.32)	658791.75 (199825.99-1184216.73)	289.86 (88.08-522.4)	-4.84 (-5.00 - -4.68)

DALYs, Disability-Adjusted Life Years; ASRs, Age-Standardized Rates; AAPC, Average Annual Percentage Change; SDI, Socio- demographic Index

Supplementary Table S2. Mortality, DALY counts, ASRs, and AAPC (%) under 14 years old across all countries and territories from 1990 to 2021.

	1990		2021		
location	Counts (95% UI)	ASR per 100,000 (95% UI)	Counts (95% UI)	ASR per 100,000 (95% UI)	AAPC in ASR from 1990 to 2021 (95% CI)
**Deaths**					
Afghanistan	804.5 (245.23-1496.74)	16.53 (5.03-30.8)	494.39 (151.6-929.68)	3.2 (0.97-6.07)	-5.15 (-5.46 - -4.83)
Albania	160.72 (55.25-273.36)	13.96 (4.83-23.85)	5.09 (1.61-8.98)	1.24 (0.39-2.22)	-7.52 (-7.90 - -7.15)
Algeria	547.67 (157.17-970.47)	5.12 (1.47-9.28)	77.68 (25.32-138.43)	0.58 (0.18-1.07)	-6.81 (-6.99 - -6.63)
American Samoa	0.88 (0.29-1.43)	4.08 (1.35-6.67)	0.21 (0.07-0.38)	1.87 (0.58-3.43)	-2.26 (-2.50 - -2.03)
Andorra	0.03 (0.01-0.07)	0.44 (0.13-0.84)	0 (0-0)	0.02 (0.01-0.04)	-9.21 (-10.15 - -8.26)
Angola	670.31 (198.48-1223.42)	12.13 (3.6-22.3)	230.39 (66.65-437.45)	1.45 (0.41-2.8)	-6.65 (-6.84 - -6.46)
Antigua and Barbuda	0.09 (0.03-0.16)	0.52 (0.16-0.9)	0.06 (0.02-0.1)	0.38 (0.12-0.68)	-0.99 (-1.49 - -0.48)
Argentina	211.8 (68.67-347.81)	2.15 (0.7-3.54)	35.19 (11.24-59.92)	0.39 (0.12-0.66)	-5.60 (-6.87 - -4.31)
Armenia	176.29 (60.61-286.23)	16.15 (5.53-26.25)	15.02 (5.41-25.39)	2.8 (1.01-4.76)	-5.40 (-6.12 - -4.69)
Australia	11.39 (4-18.83)	0.31 (0.11-0.52)	1.65 (0.51-2.89)	0.04 (0.01-0.07)	-6.59 (-8.57 - -4.57)
Austria	2.72 (0.92-4.44)	0.21 (0.07-0.34)	0.36 (0.12-0.59)	0.03 (0.01-0.05)	-6.18 (-8.01 - -4.33)
Azerbaijan	1131.12 (374.05-1922.13)	43.92 (14.47-74.82)	174.32 (55.91-300.35)	8.31 (2.67-14.41)	-5.13 (-5.43 - -4.83)
Bahamas	0.83 (0.25-1.45)	1.13 (0.33-2)	0.2 (0.06-0.38)	0.32 (0.09-0.62)	-4.14 (-5.64 - -2.62)
Bahrain	3.37 (1.08-5.83)	1.94 (0.61-3.35)	0.69 (0.25-1.23)	0.25 (0.08-0.46)	-6.50 (-7.02 - -5.97)
Bangladesh	14761.31 (5020.17-24548.52)	27.46 (9.29-45.66)	1572.96 (513.81-2714.21)	3.75 (1.21-6.62)	-6.24 (-6.83 - -5.65)
Barbados	0.32 (0.1-0.55)	0.56 (0.17-0.98)	0.07 (0.02-0.13)	0.17 (0.05-0.33)	-3.49 (-5.07 - -1.88)
Belarus	57.13 (19.27-93.6)	2.47 (0.83-4.06)	3.8 (1.29-6.54)	0.28 (0.09-0.48)	-7.03 (-7.89 - -6.17)
Belgium	6.98 (2.46-11.47)	0.4 (0.14-0.67)	1.13 (0.37-1.92)	0.06 (0.02-0.11)	-5.89 (-7.46 - -4.29)
Belize	2.62 (0.87-4.55)	3.11 (1.03-5.4)	0.71 (0.21-1.23)	0.64 (0.19-1.12)	-4.97 (-5.59 - -4.34)
Benin	380.69 (107.73-690.59)	13.62 (3.86-24.83)	134.29 (39.3-251.81)	2.03 (0.59-3.84)	-5.96 (-6.23 - -5.69)
Bermuda	0.04 (0.01-0.08)	0.34 (0.1-0.62)	0.01 (0-0.01)	0.1 (0.03-0.19)	-3.91 (-4.51 - -3.31)
Bhutan	30.34 (7.4-55.91)	11.14 (2.69-20.7)	2.38 (0.7-4.41)	1.36 (0.4-2.57)	-6.25 (-7.13 - -5.37)
Bolivia (Plurinational State of)	544.85 (171.4-977.84)	18.96 (5.9-34.07)	57.26 (16.88-104.9)	1.67 (0.48-3.13)	-7.59 (-7.92 - -7.25)
Bosnia and Herzegovina	9.67 (3.31-16.24)	0.94 (0.32-1.59)	1.07 (0.34-1.89)	0.24 (0.08-0.43)	-4.12 (-5.75 - -2.47)
Botswana	41.09 (12.92-73.14)	6.78 (2.09-12.23)	20.54 (6.79-38.42)	3.03 (0.97-5.7)	-2.69 (-3.13 - -2.25)
Brazil	4003 (1317.46-6516.79)	8.45 (2.78-13.8)	178.25 (55.25-311.3)	0.37 (0.11-0.67)	-9.70 (-10.27 - -9.13)
Brunei Darussalam	0.97 (0.33-1.64)	1.02 (0.33-1.78)	0.23 (0.07-0.41)	0.25 (0.08-0.46)	-4.51 (-5.09 - -3.94)
Bulgaria	115.15 (41.72-183.61)	7.39 (2.67-11.85)	9.74 (3.26-16.17)	1.09 (0.36-1.82)	-6.01 (-7.62 - -4.37)
Burkina Faso	1334.87 (375.1-2370.38)	25.03 (7.02-44.6)	747.99 (238.96-1388.73)	6.45 (2.01-11.92)	-4.29 (-4.67 - -3.90)
Burundi	405.99 (111.57-742.42)	13.46 (3.67-24.94)	84.01 (22.27-160.63)	1.38 (0.36-2.75)	-7.24 (-7.62 - -6.86)
Cabo Verde	4.52 (1.53-8.44)	2.68 (0.9-5.05)	0.56 (0.18-0.99)	0.42 (0.13-0.8)	-5.81 (-6.60 - -5.02)
Cambodia	2629.53 (897.69-4351.55)	50.7 (17.27-84.46)	334.95 (111.44-599.35)	6.69 (2.21-12.03)	-6.20 (-6.45 - -5.96)
Cameroon	710.5 (207.63-1263.6)	12.52 (3.62-22.35)	370.65 (108.04-713.04)	2.68 (0.78-5.18)	-4.90 (-5.15 - -4.65)
Canada	14.63 (5.03-24.96)	0.26 (0.09-0.45)	1.88 (0.57-3.37)	0.03 (0.01-0.06)	-6.21 (-7.68 - -4.72)
Central African Republic	290.15 (73.02-536.15)	20.32 (5.15-37.63)	115.4 (32.13-216.27)	4.84 (1.32-9.03)	-4.54 (-4.69 - -4.38)
Chad	661.03 (180.57-1201.73)	18.81 (5.17-34.51)	779.14 (216.96-1411.9)	7.58 (2.1-13.82)	-2.91 (-3.14 - -2.67)

	[illegible]		[illegible]		[illegible]
Chile	148.54 (51.98-235.39)	3.63 (1.27-5.75)	6.67 (2.24-10.68)	0.2 (0.07-0.33)	-8.84 (-10.87 - -6.75)
China	57036.67 (19831.07-95734.68)	17.87 (6.2-29.94)	1944.25 (670.56-3202.27)	0.85 (0.29-1.41)	-9.46 (-9.90 - -9.00)
Colombia	429.96 (142.37-741.4)	3.59 (1.18-6.19)	33.1 (9.43-64.12)	0.33 (0.09-0.65)	-7.58 (-8.30 - -6.87)
Comoros	44.33 (12.21-78.87)	18.97 (5.23-33.89)	9.2 (2.88-16.48)	3.94 (1.23-7.06)	-4.97 (-5.58 - -4.35)
Congo	58.77 (16.42-110.06)	5.24 (1.45-9.91)	18.91 (4.94-35.57)	1.03 (0.27-1.98)	-5.09 (-5.33 - -4.86)
Cook Islands	0.44 (0.13-0.74)	6.82 (2.08-11.52)	0.04 (0.01-0.08)	1.33 (0.43-2.34)	-4.85 (-6.03 - -3.65)
Costa Rica	18.08 (6.14-30.39)	1.56 (0.53-2.64)	2.08 (0.65-3.57)	0.23 (0.07-0.4)	-5.97 (-7.21 - -4.71)
Cote d'Ivoire	933.12 (267.93-1632.71)	14.25 (4.1-25.19)	512.84 (151.18-969.42)	4.15 (1.22-7.9)	-3.82 (-4.19 - -3.46)
Croatia	11.36 (4.08-18.67)	1.29 (0.46-2.1)	1.72 (0.59-2.96)	0.32 (0.11-0.56)	-4.21 (-5.34 - -3.06)
Cuba	37.77 (13.21-62.83)	1.48 (0.52-2.48)	6.52 (2.03-11)	0.41 (0.12-0.69)	-3.83 (-4.97 - -2.68)
Cyprus	0.8 (0.25-1.48)	0.43 (0.13-0.8)	0.06 (0.02-0.1)	0.03 (0.01-0.05)	-8.65 (-9.09 - -8.20)
Czechia	29.98 (9.93-48.43)	1.57 (0.52-2.58)	4.74 (1.55-8.11)	0.29 (0.09-0.5)	-5.39 (-6.13 - -4.64)
Democratic People's Republic of Korea	590.76 (191.52-1046.33)	8.93 (2.87-15.89)	58.42 (20.46-106.14)	1.33 (0.44-2.42)	-5.95 (-6.18 - -5.72)
Democratic Republic of the Congo	3014.92 (808.52-5584.49)	14.62 (3.9-27.45)	768.87 (206.71-1441.36)	1.99 (0.54-3.84)	-6.23 (-6.42 - -6.04)
Denmark	3.41 (1.15-5.69)	0.41 (0.14-0.69)	0.38 (0.12-0.64)	0.04 (0.01-0.07)	-6.81 (-7.55 - -6.06)
Djibouti	27.34 (8.93-49.77)	14.92 (4.83-27.4)	15.36 (4.37-28.87)	3.7 (1.08-7.15)	-4.37 (-4.91 - -3.82)
Dominica	0.21 (0.07-0.38)	0.86 (0.26-1.54)	0.09 (0.03-0.17)	0.87 (0.25-1.69)	0.43 (-0.23 - 1.09)
Dominican Republic	86.99 (26.75-166.1)	3.07 (0.93-5.84)	10.47 (3.45-20.76)	0.36 (0.11-0.71)	-6.70 (-7.22 - -6.17)
Ecuador	155.58 (50.86-265.59)	4.04 (1.32-6.9)	20.8 (5.94-39.22)	0.43 (0.12-0.83)	-7.02 (-7.73 - -6.31)
Egypt	5849.91 (1854.04-10019.52)	24.23 (7.63-41.85)	901.08 (290.32-1547.8)	2.42 (0.76-4.17)	-7.19 (-7.72 - -6.65)
El Salvador	47.88 (15.42-87.95)	2.18 (0.7-4.04)	5.99 (1.7-11.15)	0.34 (0.1-0.65)	-6.07 (-6.71 - -5.44)
Equatorial Guinea	58.03 (16.61-104.05)	24.96 (7.11-44.99)	11.75 (3.22-22.72)	2.14 (0.57-4.47)	-7.68 (-8.02 - -7.34)
Eritrea	183.14 (51.41-330.83)	10.41 (2.94-19.08)	66.62 (19.11-127.01)	2.56 (0.71-5)	-4.48 (-5.05 - -3.91)
Estonia	6.52 (2.22-10.55)	1.89 (0.64-3.08)	0.32 (0.11-0.55)	0.16 (0.05-0.27)	-7.75 (-9.00 - -6.47)
Eswatini	27.13 (8.03-49.12)	6.58 (1.96-11.95)	8.86 (2.73-16.77)	2.2 (0.67-4.19)	-3.56 (-4.07 - -3.04)
Ethiopia	2701.63 (754.78-4840.2)	9.91 (2.77-17.78)	495.21 (150.62-925.68)	1.09 (0.33-2.05)	-7.05 (-7.41 - -6.69)
Fiji	11.13 (3.32-19.29)	4.1 (1.21-7.22)	5.23 (1.55-9.57)	1.99 (0.58-3.73)	-2.35 (-2.67 - -2.02)
Finland	2.26 (0.78-3.79)	0.25 (0.08-0.42)	0.3 (0.1-0.52)	0.04 (0.01-0.07)	-5.95 (-7.87 - -4.00)
France	32.18 (10.98-51.49)	0.29 (0.1-0.46)	5.87 (2.08-9.7)	0.06 (0.02-0.09)	-5.19 (-6.49 - -3.87)
Gabon	27.5 (7.78-52.62)	6.21 (1.75-11.82)	7.87 (2.28-15.22)	1.28 (0.37-2.46)	-4.96 (-5.20 - -4.71)
Gambia	90.4 (29.05-160.34)	17.23 (5.51-30.74)	24.74 (8.06-44.91)	2.44 (0.78-4.5)	-6.23 (-7.23 - -5.22)
Georgia	234.27 (80.75-389.83)	17.47 (6.02-29.15)	7.13 (2.43-12.16)	1.01 (0.34-1.74)	-8.82 (-9.88 - -7.75)
Germany	40.16 (14.1-65.48)	0.31 (0.11-0.52)	3.6 (1.17-5.91)	0.03 (0.01-0.05)	-7.14 (-8.12 - -6.15)
Ghana	305.88 (85.61-569.07)	4.1 (1.14-7.72)	113.07 (29.22-220.93)	0.86 (0.22-1.71)	-4.84 (-5.21 - -4.47)
Greece	5.5 (1.94-8.71)	0.32 (0.11-0.52)	1.15 (0.39-1.91)	0.09 (0.03-0.15)	-3.75 (-5.58 - -1.88)
Greenland	0.41 (0.14-0.69)	2.61 (0.87-4.41)	0.03 (0.01-0.05)	0.27 (0.09-0.49)	-6.28 (-6.79 - -5.77)
Grenada	0.55 (0.18-0.96)	1.59 (0.52-2.82)	0.1 (0.03-0.18)	0.49 (0.14-0.89)	-3.35 (-4.87 - -1.81)
Guam	0.96 (0.33-1.64)	2.12 (0.72-3.6)	0.42 (0.13-0.76)	1.16 (0.35-2.09)	-2.02 (-2.31 - -1.74)
	[illegible]		[illegible]		[illegible]

Guatemala	385.31 (125.97-663.97)	8.91 (2.89-15.41)	124.73 (37.2-224.81)	2.77 (0.83-4.97)	-3.81 (-4.39 - -3.23)
Guinea	1014.75 (284.93-1860.67)	30.84 (8.7-56.76)	345.83 (96.3-643.24)	5.41 (1.51-10.09)	-5.46 (-5.89 - -5.02)
Guinea-Bissau	39.68 (10.39-77.05)	7.44 (1.95-14.54)	12.77 (3.27-23.87)	1.36 (0.36-2.61)	-5.31 (-5.83 - -4.78)
Guyana	9.23 (2.83-15.49)	2.89 (0.89-4.88)	2.11 (0.61-3.91)	0.99 (0.29-1.86)	-3.45 (-4.88 - -2.00)
Haiti	474.6 (140-877.81)	15.74 (4.65-29.27)	126.12 (38.93-232.29)	2.82 (0.87-5.27)	-5.37 (-5.58 - -5.16)
Honduras	144.45 (44.55-256.3)	6.18 (1.9-11.05)	26.22 (8.05-48.67)	0.83 (0.25-1.58)	-6.28 (-6.59 - -5.97)
Hungary	24.28 (8.33-38.98)	1.35 (0.46-2.16)	2.05 (0.64-3.7)	0.16 (0.05-0.28)	-6.48 (-7.67 - -5.26)
Iceland	0.21 (0.07-0.35)	0.34 (0.12-0.58)	0.04 (0.01-0.07)	0.06 (0.02-0.12)	-5.26 (-5.61 - -4.90)
India	59847.76 (19348.17-103598.8)	18.02 (5.81-31.22)	11052.67 (3347.91-19538.37)	3.44 (1.04-6.13)	-5.41 (-5.69 - -5.13)
Indonesia	8402.61 (2809.12-13719.03)	13.13 (4.37-21.68)	1462.69 (493.43-2542.66)	2.32 (0.78-4.04)	-5.34 (-5.54 - -5.14)
Iran (Islamic Republic of)	892.98 (275.6-1605.64)	3.55 (1.11-6.43)	35.71 (12.45-60.86)	0.18 (0.06-0.32)	-9.00 (-9.65 - -8.34)
Iraq	741.66 (254.06-1296.04)	8.34 (2.83-14.45)	152.87 (46.88-265.63)	1.23 (0.38-2.17)	-6.09 (-6.45 - -5.74)
Ireland	4.66 (1.57-7.6)	0.54 (0.18-0.88)	0.56 (0.18-0.95)	0.06 (0.02-0.11)	-6.63 (-7.75 - -5.50)
Israel	5.63 (1.91-9.07)	0.38 (0.13-0.61)	0.67 (0.21-1.15)	0.03 (0.01-0.04)	-8.29 (-9.69 - -6.87)
Italy	34.55 (12-55.98)	0.43 (0.15-0.69)	2.52 (0.82-4.25)	0.04 (0.01-0.07)	-7.60 (-9.17 - -6.01)
Jamaica	8.83 (2.78-14.9)	1.1 (0.34-1.85)	1.17 (0.35-2.22)	0.23 (0.07-0.44)	-4.89 (-5.92 - -3.85)
Japan	97.48 (34.83-156.59)	0.48 (0.17-0.78)	9.05 (3.02-15.15)	0.06 (0.02-0.11)	-6.28 (-7.39 - -5.15)
Jordan	95.2 (31.81-157.31)	5.59 (1.87-9.28)	31.44 (10.62-52.87)	0.97 (0.33-1.63)	-5.52 (-5.84 - -5.20)
Kazakhstan	804.02 (266.43-1327.99)	14.98 (4.96-24.75)	63 (21.74-104.67)	1.14 (0.39-1.89)	-7.86 (-8.78 - -6.93)
Kenya	1218.19 (399.29-2140.05)	10 (3.27-17.62)	252.32 (78.23-455.39)	1.46 (0.44-2.66)	-6.22 (-6.53 - -5.91)
Kiribati	4.93 (1.68-8.2)	14.78 (4.99-24.73)	1.83 (0.64-3.2)	4.46 (1.54-7.94)	-3.79 (-4.18 - -3.39)
Kuwait	12.74 (4.28-21.07)	2.21 (0.74-3.69)	7.76 (2.61-12.74)	0.99 (0.33-1.65)	-1.17 (-4.05 - 1.80)
Kyrgyzstan	486.65 (163.52-791.41)	26.63 (8.9-43.31)	56.37 (19.52-92.85)	2.48 (0.86-4.11)	-7.22 (-8.31 - -6.12)
Lao People's Democratic Republic	1141.96 (367.4-1952.78)	56.54 (18.17-97.02)	179.86 (53.7-327.94)	7.62 (2.24-13.8)	-5.83 (-6.06 - -5.60)
Latvia	10 (3.48-16.05)	1.73 (0.6-2.79)	0.7 (0.24-1.17)	0.26 (0.09-0.44)	-5.91 (-7.31 - -4.48)
Lebanon	56.61 (15.97-98.79)	5.13 (1.46-9.13)	10.77 (3.28-18.85)	0.9 (0.27-1.6)	-5.50 (-5.77 - -5.23)
Lesotho	63.38 (19.83-109.15)	9.03 (2.79-15.77)	55.32 (16.38-99.78)	9.41 (2.77-17.17)	0.04 (-0.47 - 0.56)
Liberia	228.96 (67.88-432.91)	17.39 (5.12-33.06)	31.16 (8.2-58.37)	1.42 (0.37-2.7)	-7.77 (-8.60 - -6.92)
Libya	52.21 (17.45-92.9)	2.87 (0.94-5.18)	11.45 (3.7-19.98)	0.89 (0.26-1.64)	-3.39 (-4.72 - -2.03)
Lithuania	9.79 (3.5-15.68)	1.19 (0.43-1.92)	0.88 (0.3-1.48)	0.23 (0.08-0.39)	-5.26 (-6.63 - -3.86)
Luxembourg	0.19 (0.06-0.32)	0.29 (0.09-0.49)	0.04 (0.01-0.06)	0.04 (0.01-0.07)	-6.76 (-8.38 - -5.12)
Madagascar	1673.06 (551.37-2860.68)	27.54 (9.08-47.28)	495.68 (150.31-917.98)	4.24 (1.28-7.83)	-5.93 (-6.16 - -5.69)
Malawi	871.16 (246.76-1612.21)	16.28 (4.6-30.19)	196.69 (62.83-367.05)	2.51 (0.79-4.78)	-5.89 (-6.58 - -5.19)
Malaysia	187.19 (58.93-323.2)	2.78 (0.87-4.85)	42.92 (15.28-70.86)	0.59 (0.2-1.03)	-4.96 (-6.01 - -3.89)
Maldives	10.58 (3.59-18.26)	9.02 (3.06-15.59)	0.82 (0.29-1.39)	0.88 (0.31-1.52)	-7.32 (-7.89 - -6.73)
Mali	481.92 (130.75-877.47)	9.86 (2.67-18.15)	512.91 (154.93-958.32)	3.96 (1.18-7.46)	-2.91 (-3.29 - -2.53)
Malta	0.37 (0.12-0.61)	0.45 (0.15-0.75)	0.07 (0.02-0.13)	0.11 (0.03-0.2)	-4.31 (-5.56 - -3.05)
Marshall Islands	1.57 (0.5-2.77)	7.38 (2.31-13.07)	0.67 (0.22-1.19)	4.11 (1.35-7.3)	-1.88 (-2.17 - -1.60)
Mauritania	148.16 (48.94-256.29)	14.13 (4.65-24.73)	51.94 (16.71-91.91)	2.77 (0.88-4.96)	-5.05 (-5.41 - -4.68)

Mauritius	8.57 (2.96-13.91)	2.79 (0.97-4.52)	2.13 (0.69-3.62)	1.13 (0.37-1.94)	-2.91 (-4.36 - -1.43)
Mexico	2884.24 (973.79-4754.95)	8.56 (2.89-14.1)	136.57 (40.13-253.94)	0.47 (0.14-0.89)	-8.84 (-9.59 - -8.08)
Micronesia (Federated States of)	5.99 (2.06-10.12)	13.35 (4.57-22.61)	0.94 (0.3-1.64)	3.39 (1.06-5.97)	-4.32 (-4.69 - -3.96)
Monaco	0.01 (0-0.02)	0.36 (0.11-0.66)	0.01 (0-0.01)	0.12 (0.04-0.23)	-3.04 (-3.86 - -2.21)
Mongolia	394.2 (132.55-652.22)	40.74 (13.65-67.46)	35.68 (11.86-62.11)	3.21 (1.07-5.63)	-7.80 (-8.35 - -7.24)
Montenegro	2.49 (0.82-4.32)	1.65 (0.53-2.91)	0.16 (0.05-0.28)	0.15 (0.05-0.27)	-7.28 (-8.15 - -6.41)
Morocco	1316.65 (415.05-2224.35)	12.97 (4.05-22.04)	81.82 (26.54-151.67)	0.87 (0.28-1.64)	-8.35 (-8.48 - -8.23)
Mozambique	1211.56 (348.07-2214.32)	17.72 (5.08-32.71)	353.94 (99.09-642.31)	2.41 (0.68-4.47)	-6.36 (-7.04 - -5.67)
Myanmar	6877.49 (2257.3-11840.6)	47.68 (15.51-82.56)	828.76 (254.29-1414.97)	5.53 (1.68-9.51)	-6.76 (-6.90 - -6.62)
Namibia	54.29 (18.25-94.19)	8.47 (2.82-14.96)	19.15 (6.32-35.63)	2.39 (0.77-4.49)	-4.14 (-4.43 - -3.84)
Nauru	0.53 (0.18-0.9)	11.4 (3.77-19.7)	0.28 (0.1-0.49)	7.03 (2.34-12.41)	-1.57 (-1.81 - -1.33)
Nepal	4460.51 (1434.11-7367.16)	47.57 (15.32-78.82)	291.79 (90.04-548.35)	3.28 (1.01-6.24)	-8.27 (-8.63 - -7.90)
Netherlands	9.6 (3.31-15.36)	0.36 (0.12-0.57)	1.28 (0.41-2.21)	0.05 (0.02-0.09)	-5.84 (-6.92 - -4.75)
New Zealand	6.14 (2.14-10.16)	0.77 (0.27-1.28)	0.82 (0.26-1.41)	0.09 (0.03-0.16)	-7.17 (-9.09 - -5.21)
Nicaragua	156.75 (50.05-274.58)	8.28 (2.64-14.53)	16.77 (5.02-30.43)	0.9 (0.27-1.64)	-7.02 (-7.63 - -6.41)
Niger	1367.01 (403.1-2527.43)	28.54 (8.33-53.03)	562.67 (155.91-1034.12)	3.9 (1.08-7.2)	-6.20 (-6.78 - -5.61)
Nigeria	5803.09 (1706.58-10385.09)	12.88 (3.76-23.12)	2850.9 (807.95-5343.2)	2.7 (0.76-5.07)	-4.95 (-5.22 - -4.69)
Niue	0.04 (0.01-0.07)	5.02 (1.55-8.95)	0.04 (0.01-0.06)	10.29 (3.55-17.54)	2.72 (1.24 - 4.23)
North Macedonia	32.59 (10.37-55.86)	6.67 (2.12-11.47)	0.8 (0.26-1.38)	0.27 (0.09-0.48)	-9.81 (-10.95 - -8.67)
Northern Mariana Islands	0.26 (0.09-0.46)	1.93 (0.67-3.46)	0.06 (0.02-0.11)	0.66 (0.22-1.16)	-3.45 (-4.59 - -2.29)
Norway	2.25 (0.82-3.69)	0.29 (0.1-0.47)	0.18 (0.06-0.31)	0.02 (0.01-0.04)	-8.48 (-10.20 - -6.72)
Oman	30.3 (9.63-55.78)	3.28 (1.04-6.11)	5.2 (1.64-9.29)	0.43 (0.13-0.78)	-6.59 (-7.36 - -5.82)
Pakistan	9895.96 (3203.42-16413.81)	18.8 (6.08-31.19)	3990.89 (1291.69-7104.92)	4.7 (1.53-8.36)	-4.43 (-4.86 - -4.00)
Palau	0.26 (0.08-0.46)	6.1 (1.99-10.96)	0.06 (0.02-0.1)	2.09 (0.64-3.54)	-3.36 (-3.61 - -3.11)
Palestine	57.54 (19.51-100.73)	5.26 (1.77-9.29)	14.56 (4.8-24.84)	0.81 (0.26-1.41)	-5.80 (-6.19 - -5.41)
Panama	13.47 (4.23-23.47)	1.65 (0.52-2.88)	4.91 (1.48-8.93)	0.46 (0.14-0.83)	-4.21 (-5.20 - -3.21)
Papua New Guinea	645.61 (216.37-1084.49)	34.95 (11.69-58.89)	756.01 (263.7-1326.66)	17.45 (5.99-30.89)	-2.24 (-2.51 - -1.97)
Paraguay	64.58 (20.86-113.79)	3.68 (1.18-6.49)	8.74 (2.51-17.27)	0.46 (0.13-0.93)	-6.67 (-7.79 - -5.53)
Peru	770.54 (242.51-1358.3)	9.22 (2.89-16.28)	57.12 (16.5-107.76)	0.6 (0.17-1.16)	-8.66 (-9.75 - -7.55)
Philippines	4515.62 (1473.82-7621.67)	17.28 (5.68-29.11)	936.12 (310.28-1608.63)	2.88 (0.94-5.03)	-5.75 (-6.21 - -5.28)
Poland	111.67 (39.51-176.04)	1.31 (0.46-2.07)	10.84 (3.58-18.2)	0.19 (0.06-0.33)	-5.85 (-7.85 - -3.81)
Portugal	18.07 (6.24-30.07)	1.03 (0.35-1.72)	1.16 (0.37-1.93)	0.09 (0.03-0.15)	-7.63 (-9.71 - -5.50)
Puerto Rico	3.91 (1.21-6.71)	0.42 (0.13-0.73)	0.27 (0.08-0.48)	0.09 (0.03-0.15)	-4.78 (-5.52 - -4.04)
Qatar	1.72 (0.51-3.2)	1.22 (0.36-2.26)	0.8 (0.27-1.39)	0.15 (0.05-0.28)	-6.45 (-6.85 - -6.06)
Republic of Korea	133.18 (45.76-224.81)	1.31 (0.45-2.24)	2.38 (0.78-4.12)	0.04 (0.01-0.08)	-10.26 (-10.80 - -9.72)
Republic of Moldova	86.64 (29.91-144.28)	7.04 (2.42-11.71)	5.66 (1.95-9.8)	1.26 (0.43-2.18)	-5.29 (-6.40 - -4.17)
Romania	666.9 (229.62-1087.85)	13.12 (4.51-21.44)	35.41 (12.1-58.88)	1.29 (0.43-2.14)	-6.91 (-8.02 - -5.80)
Russian Federation	1251.74 (446.89-2002.82)	3.76 (1.34-6.01)	105.11 (36.83-174.5)	0.46 (0.16-0.77)	-6.43 (-7.39 - -5.46)
Rwanda	610.72 (174.02-1116.01)	16.05 (4.51-29.74)	84.59 (23.21-156.61)	1.7 (0.46-3.21)	-6.97 (-8.09 - -5.83)

[illegible]	[illegible]		[illegible]		[illegible]
Saint Kitts and Nevis	0.16 (0.05-0.27)	1.15 (0.37-2.01)	0.04 (0.01-0.08)	0.5 (0.15-0.94)	-2.55 (-3.52 - -1.57)
Saint Lucia	0.43 (0.14-0.77)	0.86 (0.27-1.53)	0.08 (0.02-0.15)	0.31 (0.09-0.6)	-3.16 (-3.91 - -2.40)
Saint Vincent and the Grenadines	0.47 (0.15-0.86)	1.28 (0.41-2.33)	0.09 (0.03-0.16)	0.39 (0.12-0.73)	-3.61 (-4.37 - -2.84)
Samoa	5.55 (1.78-9.74)	7.64 (2.42-13.43)	2.11 (0.63-3.94)	2.54 (0.74-4.77)	-3.48 (-3.78 - -3.18)
San Marino	0.01 (0-0.02)	0.32 (0.1-0.57)	0 (0-0)	0.02 (0.01-0.04)	-8.21 (-8.53 - -7.89)
Sao Tome and Principe	2.24 (0.61-4.19)	3.84 (1.05-7.18)	0.22 (0.06-0.45)	0.3 (0.08-0.62)	-7.76 (-8.73 - -6.77)
Saudi Arabia	236.83 (70.75-430.31)	3.45 (1.04-6.33)	12.79 (4.28-23.9)	0.18 (0.06-0.34)	-9.23 (-9.47 - -8.99)
Senegal	788.69 (244-1355.37)	19.01 (5.85-32.78)	151.45 (45.46-277.27)	2.34 (0.71-4.36)	-6.49 (-7.45 - -5.52)
Serbia	60 (20.89-107.77)	3.05 (1.04-5.53)	2.08 (0.7-3.44)	0.19 (0.06-0.32)	-7.87 (-10.37 - -5.30)
Seychelles	0.74 (0.24-1.23)	3.18 (1.06-5.4)	0.4 (0.12-0.69)	1.77 (0.54-3.11)	-1.80 (-2.69 - -0.90)
Sierra Leone	874 (258.87-1573.41)	39.91 (11.79-71.76)	183.97 (54.03-348.64)	4.86 (1.42-9.42)	-6.58 (-7.12 - -6.03)
Singapore	9.69 (3.19-16.56)	1.61 (0.53-2.78)	1.05 (0.34-1.84)	0.13 (0.04-0.23)	-7.75 (-9.47 - -6.01)
Slovakia	42.23 (14.45-68.55)	3.54 (1.2-5.82)	5.48 (1.81-9.45)	0.66 (0.22-1.16)	-5.33 (-6.62 - -4.02)
Slovenia	5.78 (2.05-9.46)	1.6 (0.56-2.63)	0.79 (0.27-1.33)	0.27 (0.09-0.46)	-5.45 (-6.31 - -4.57)
Solomon Islands	30.07 (10.01-50.08)	17.76 (5.95-29.81)	18.57 (6.06-31.97)	6.86 (2.19-11.9)	-3.06 (-3.62 - -2.49)
Somalia	813.48 (216.78-1493.41)	18.57 (4.94-34.4)	578.59 (154.86-1106.16)	4.98 (1.33-9.56)	-4.08 (-4.53 - -3.63)
South Africa	2159.79 (705.57-3682.46)	15.53 (5.07-26.53)	427.47 (144.04-737.22)	2.97 (1-5.2)	-5.36 (-5.67 - -5.05)
South Sudan	548.94 (168.95-1018.7)	18.98 (5.83-35.32)	313.3 (96.2-596.87)	7.06 (2.17-13.57)	-3.23 (-3.73 - -2.73)
Spain	26.02 (9.05-41.07)	0.41 (0.14-0.64)	2.55 (0.88-4.27)	0.04 (0.01-0.07)	-6.97 (-8.47 - -5.44)
Sri Lanka	101.8 (35.66-170.91)	1.96 (0.67-3.29)	12.84 (3.49-24.28)	0.27 (0.07-0.52)	-6.15 (-7.45 - -4.82)
Sudan	1723.57 (520.99-3211.94)	17.41 (5.2-32.57)	223.58 (64.86-411.82)	1.38 (0.4-2.59)	-7.84 (-8.05 - -7.64)
Suriname	5.21 (1.8-8.95)	4.15 (1.42-7.16)	1.35 (0.4-2.53)	1.05 (0.31-1.98)	-4.35 (-5.08 - -3.61)
Sweden	2.8 (0.93-4.66)	0.18 (0.06-0.3)	0.28 (0.09-0.48)	0.02 (0-0.03)	-7.63 (-8.99 - -6.25)
Switzerland	4.08 (1.46-6.71)	0.36 (0.13-0.59)	0.47 (0.15-0.79)	0.04 (0.01-0.06)	-7.07 (-8.75 - -5.36)
Syrian Arab Republic	398.93 (124.47-714.65)	6.51 (2-11.83)	30.24 (8.8-54.38)	0.92 (0.26-1.71)	-6.24 (-7.17 - -5.31)
Taiwan (Province of China)	81.07 (27.25-131.9)	1.7 (0.57-2.78)	8.83 (2.96-15.22)	0.34 (0.11-0.58)	-5.09 (-6.61 - -3.54)
Tajikistan	823.96 (274.5-1426.54)	30.75 (10.25-53.45)	208.37 (60.73-376.71)	5.48 (1.59-10.02)	-5.27 (-5.49 - -5.05)
Thailand	659.66 (230.42-1105.27)	4.37 (1.45-7.49)	70.17 (23.31-119.47)	0.82 (0.27-1.42)	-5.24 (-5.64 - -4.83)
Timor-Leste	216.34 (73.08-371.06)	54.53 (18.47-93.74)	46.72 (16.25-81.04)	8.86 (3.07-15.48)	-5.62 (-5.95 - -5.28)
Togo	189.6 (57.37-336.33)	9.75 (2.93-17.47)	63.12 (16.53-118.65)	1.89 (0.49-3.57)	-5.23 (-5.51 - -4.94)
Tokelau	0.04 (0.01-0.07)	6.88 (2.08-12.15)	0.04 (0.01-0.06)	12.64 (4.49-21.51)	3.13 (1.31 - 4.97)
Tonga	2.8 (0.94-4.76)	6.45 (2.14-11.07)	1.06 (0.34-1.91)	2.59 (0.81-4.67)	-2.86 (-3.26 - -2.47)
Trinidad and Tobago	5.92 (1.85-9.85)	1.54 (0.48-2.56)	1.03 (0.31-1.86)	0.43 (0.13-0.79)	-4.02 (-4.64 - -3.40)
Tunisia	272.81 (86.15-553.58)	8.94 (2.81-18.33)	17.86 (6.15-34.23)	0.69 (0.23-1.37)	-7.96 (-8.31 - -7.60)
Turkey	3376.32 (1197.02-5909.38)	16.96 (5.98-29.82)	99.77 (32.93-172.04)	0.6 (0.2-1.05)	-10.23 (-10.59 - -9.86)
Turkmenistan	564.57 (184.74-942.48)	33.91 (11.11-56.59)	97.75 (32.09-167.91)	6.35 (2.08-11.01)	-5.14 (-5.87 - -4.40)
Tuvalu	1.24 (0.41-2.18)	29.06 (9.47-51.26)	0.15 (0.05-0.26)	4.19 (1.4-7.27)	-6.05 (-6.30 - -5.81)
Uganda	741.13 (213.9-1381.52)	7.34 (2.1-14.01)	292.05 (76.92-556.35)	1.42 (0.36-2.72)	-5.22 (-5.85 - -4.58)
Ukraine	267.56 (96.77-439.38)	2.47 (0.89-4.08)	22.73 (7.57-37.78)	0.46 (0.16-0.78)	-5.05 (-6.62 - -3.45)
United Arab Emirates	11.48 (3.96-20.15)	1.8 (0.61-3.22)	2.67 (0.94-4.57)	0.21 (0.07-0.38)	-6.54 (-7.28 - -5.80)

United Kingdom	48.05 (17-79.31)	0.44 (0.15-0.72)	7.16 (2.24-12.27)	0.07 (0.02-0.12)	-5.95 (-7.61 - -4.25)
United Republic of Tanzania	3639.38 (1156.79-6386.13)	26.72 (8.5-47)	691.02 (201.86-1288.51)	2.75 (0.79-5.14)	-7.17 (-7.49 - -6.86)
United States of America	203.18 (69.14-334.84)	0.36 (0.12-0.6)	35.61 (11.45-61.23)	0.06 (0.02-0.11)	-5.33 (-6.22 - -4.44)
United States Virgin Islands	0.15 (0.04-0.26)	0.46 (0.13-0.85)	0.01 (0-0.01)	0.07 (0.02-0.13)	-5.99 (-6.31 - -5.68)
Uruguay	15.12 (5.3-24.47)	1.93 (0.68-3.14)	1.71 (0.54-2.9)	0.29 (0.09-0.51)	-5.53 (-6.42 - -4.64)
Uzbekistan	1307.84 (414.59-2236.04)	13.65 (4.33-23.38)	515.01 (170.28-872.47)	4.75 (1.55-8.12)	-3.17 (-3.61 - -2.74)
Vanuatu	5.35 (1.64-9.85)	7 (2.16-13.06)	2.98 (0.89-5.31)	2.49 (0.73-4.5)	-3.10 (-3.95 - -2.25)
Venezuela (Bolivarian Republic of)	238.92 (81.81-394.84)	3.31 (1.14-5.48)	60.24 (18.99-109.13)	0.95 (0.3-1.74)	-3.70 (-5.15 - -2.22)
Viet Nam	3004.87 (1129.08-5006.78)	11.18 (4.18-18.68)	356.45 (112.11-622.44)	1.52 (0.47-2.68)	-6.22 (-6.50 - -5.93)
Yemen	1473.7 (464.18-2691.82)	18.66 (5.83-33.98)	353.6 (121.61-633.12)	2.62 (0.86-4.73)	-6.13 (-6.47 - -5.78)
Zambia	943.81 (290.63-1699.57)	22.06 (6.74-39.75)	237.91 (68.37-432.51)	2.85 (0.83-5.22)	-6.42 (-6.71 - -6.13)
Zimbabwe	395.01 (119.1-712.18)	7.86 (2.36-14.25)	344.35 (105.11-604.63)	5.46 (1.66-9.59)	-1.02 (-1.62 - -0.42)
**DALYs**					
Afghanistan	71578.81 (21819.72-133018.87)	1469.94 (447.6-2736.99)	43854.65 (13445.18-82368.5)	283.45 (85.8-537.79)	-5.16 (-5.47 - -4.84)
Albania	14316.2 (4925.82-24350.87)	1243.23 (430.16-2123.59)	450.5 (142.86-796.14)	109.59 (34.46-196.5)	-7.53 (-7.91 - -7.16)
Algeria	48594.51 (13893.28-86286.66)	454.72 (130.13-823.05)	6833.72 (2217.14-12181.69)	50.96 (15.85-93.98)	-6.84 (-7.01 - -6.67)
American Samoa	77.97 (25.73-126.65)	362.77 (119.72-591.97)	18.35 (5.8-33.44)	165.48 (51.63-303.29)	-2.27 (-2.51 - -2.03)
Andorra	3.07 (0.94-5.74)	38.81 (11.81-73.83)	0.16 (0.05-0.28)	1.89 (0.6-3.59)	-9.20 (-10.14 - -8.25)
Angola	59315.21 (17590.52-108266.21)	1072.22 (318.84-1971.14)	20313.83 (5880.53-38586.32)	127.34 (36.28-246.2)	-6.66 (-6.85 - -6.48)
Antigua and Barbuda	8.06 (2.54-13.86)	46.31 (14.5-80.17)	5.12 (1.63-9.17)	33.56 (10.62-60.22)	-1.00 (-1.50 - -0.49)
Argentina	18870.42 (6124.62-30995.41)	191.7 (62.24-315.58)	3082.28 (983.1-5257.9)	34.1 (10.79-58.05)	-5.64 (-6.92 - -4.35)
Armenia	15731.36 (5408.53-25545.04)	1440.55 (493.67-2341.93)	1334.87 (480.67-2257.92)	248.7 (89.64-423.63)	-5.41 (-6.12 - -4.70)
Australia	1014.08 (356.59-1677.83)	27.95 (9.89-46.53)	146.6 (45.15-256.42)	3.32 (1.03-5.87)	-6.59 (-8.55 - -4.59)
Austria	242.13 (81.71-396.37)	18.8 (6.33-30.75)	32.03 (10.73-53.21)	2.56 (0.87-4.28)	-6.14 (-7.97 - -4.28)
Azerbaijan	100923.87 (33371.68-171553.99)	3917.61 (1291.07-6675)	15463.57 (4957.14-26643.13)	738.12 (236.94-1280.09)	-5.14 (-5.44 - -4.85)
Bahamas	74.3 (21.97-129.36)	101.11 (29.79-177.93)	18.08 (5.25-34.05)	28.79 (8.28-55.08)	-4.15 (-5.67 - -2.62)
Bahrain	299.59 (96.25-518.22)	171.99 (54.05-297.23)	59.9 (21.59-108.4)	21.46 (7.44-39.75)	-6.54 (-7.07 - -6.00)
Bangladesh	1313538.66 (446889.56-2183996.51)	2442.61 (826.79-4059.1)	139012.36 (45313.96-240361.97)	332.39 (107.43-585.98)	-6.26 (-6.84 - -5.67)
Barbados	28.12 (8.89-49.33)	49.57 (15.48-87.63)	6.11 (1.71-11.61)	15.33 (4.25-29.25)	-3.48 (-5.05 - -1.88)
Belarus	5116.12 (1726.79-8381.39)	221.67 (74.63-364.36)	341.97 (116.79-586.85)	24.83 (8.35-42.97)	-7.02 (-7.88 - -6.15)
Belgium	616.09 (217.19-1012.58)	35.75 (12.47-59.5)	99.11 (32.19-169.02)	5.61 (1.8-9.85)	-5.79 (-7.51 - -4.04)
Belize	232.86 (77.68-404.81)	276.38 (91.85-480.99)	62.88 (18.31-109.64)	57.02 (16.52-99.91)	-4.97 (-5.59 - -4.34)
Benin	33672.92 (9532.07-60998.18)	1203.53 (341.37-2190.71)	11861.05 (3472.52-22256.44)	178.98 (52.13-338.19)	-5.96 (-6.23 - -5.69)
Bermuda	3.75 (1.16-6.74)	30.62 (9.42-55.34)	0.67 (0.2-1.23)	8.99 (2.66-16.89)	-3.87 (-4.41 - -3.32)
Bhutan	2702.76 (659.84-4979.11)	992.16 (240.2-1842.18)	211.77 (61.92-392.9)	120.8 (35.33-228.46)	-6.26 (-7.12 - -5.38)
Bolivia [illegible]	48414.24 [illegible]	1684.28 [illegible]	5064.41 [illegible]	148.02 [illegible]	-7.60 (-7.93 - [illegible]

(Plurinational State of)	(15221.4-86865.43)	[illegible] (523.84-3025.78)	[illegible] (1490.25-9296.02)	[illegible] (42.55-277.16)	[illegible] -7.26)
Bosnia and Herzegovina	856.7 (293.62-1438.69)	83.65 (28.68-141.12)	94.79 (30.42-167.12)	21.04 (6.79-38.13)	-4.11 (-5.60 - -2.60)
Botswana	3632.34 (1140.49-6464.5)	599.27 (185.02-1079.42)	1807.78 (596.4-3384.84)	267.16 (85.24-502.18)	-2.70 (-3.13 - -2.27)
Brazil	356622.02 (117342.65-580788.06)	753.64 (247.74-1229.47)	15746.49 (4884.13-27484.61)	33.13 (10.12-58.89)	-9.73 (-10.28 - -9.16)
Brunei Darussalam	84.98 (29.06-143.8)	88.76 (29.16-155.4)	19.99 (6.32-36.52)	22.29 (6.9-41.22)	-4.46 (-5.05 - -3.86)
Bulgaria	10203.26 (3695.62-16277.54)	655.92 (236.98-1051.82)	855.84 (286.77-1421.5)	96.12 (32.05-160.21)	-6.09 (-7.84 - -4.30)
Burkina Faso	118414.77 (33281.96-210513.92)	2219.09 (622.87-3961.09)	66262.84 (21156.55-122955.08)	570.49 (178.33-1053.09)	-4.29 (-4.67 - -3.91)
Burundi	35884.46 (9848.21-65597.62)	1188.04 (323.44-2198.67)	7379.67 (1960.52-14110.92)	121.21 (31.35-240.89)	-7.17 (-7.53 - -6.81)
Cabo Verde	402.27 (135.93-751.79)	238.1 (80.22-448.57)	48.62 (15.87-86.53)	37.16 (11.51-70.46)	-5.85 (-6.62 - -5.07)
Cambodia	233905.02 (79936.12-387179.29)	4507.91 (1537.31-7506.47)	29744.56 (9894.64-53268.36)	594.03 (196.02-1068.81)	-6.20 (-6.45 - -5.96)
Cameroon	62924.52 (18381.59-111815.71)	1107.65 (320.87-1976.15)	32739.27 (9549.12-62974.7)	236.32 (68.78-457.29)	-4.91 (-5.16 - -4.66)
Canada	1290.17 (443.6-2196.82)	23.19 (7.95-39.6)	165.93 (50.75-297.68)	2.91 (0.86-5.24)	-6.20 (-7.64 - -4.74)
Central African Republic	25784 (6486.86-47637.45)	1804.71 (456.75-3340.84)	10209.82 (2842.05-19111.11)	428.07 (117.33-797.99)	-4.55 (-4.70 - -4.39)
Chad	58509.11 (16016.77-106380.48)	1663.09 (458.06-3049.47)	68865.17 (19184.87-124594.36)	669.52 (185.75-1220.09)	-2.91 (-3.14 - -2.68)
Chile	13259 (4641.43-21006.84)	323.67 (113.28-512.95)	584.22 (196.26-936.79)	17.75 (5.89-28.94)	-9.12 (-10.97 - -7.24)
China	5074863.14 (1765111.87-8517602.27)	1589.61 (551.9-2664.19)	173236.45 (59771.42-285452.04)	75.98 (26.18-125.73)	-9.45 (-9.89 - -9.00)
Colombia	38217.03 (12654.9-65943.84)	318.97 (105.34-549.98)	2915.24 (831.3-5649.28)	29.11 (8.33-57.55)	-7.51 (-8.21 - -6.81)
Comoros	3941.77 (1087.15-7010.58)	1685.43 (465.27-3009.58)	811.58 (254.41-1453.57)	347.66 (108.25-623.55)	-4.99 (-5.59 - -4.38)
Congo	5208.42 (1456.18-9745.92)	464.53 (128.68-876.79)	1660.25 (433.25-3124.78)	90.42 (23.44-173.97)	-5.12 (-5.35 - -4.88)
Cook Islands	38.88 (11.81-65.29)	605.43 (184.55-1021)	3.88 (1.23-6.76)	117.49 (38.03-206.54)	-4.86 (-6.04 - -3.67)
Costa Rica	1612.45 (547.85-2710.95)	139.37 (47.43-235.23)	184.08 (57.29-316.31)	20.38 (6.31-35.79)	-5.98 (-7.23 - -4.72)
Cote d'Ivoire	82928.92 (23859.27-145310.2)	1265.21 (364.98-2238.06)	45428.81 (13408.16-85998.55)	367.62 (108.52-699.57)	-3.83 (-4.20 - -3.47)
Croatia	1005.73 (360.91-1653.2)	114.22 (40.74-186.06)	152.53 (52.04-262.15)	28.6 (9.78-49.47)	-4.20 (-5.34 - -3.03)
Cuba	3364.9 (1177.85-5607.02)	131.99 (46.08-220.6)	578.64 (179.62-974.63)	36.31 (11.12-61.07)	-3.84 (-4.98 - -2.68)
Cyprus	70.99 (22.31-131.25)	38.56 (11.98-71.52)	5.3 (1.77-9.17)	2.45 (0.79-4.32)	-8.64 (-9.06 - -8.22)
Czechia	2650.91 (877.22-4289.5)	139.34 (46.45-228.44)	416.3 (135.78-713.58)	25.42 (8.18-43.74)	-5.41 (-6.15 - -4.66)
Democratic People's Republic of Korea	52683.86 (17081.67-93312.26)	795.9 (255.54-1415.68)	5178.39 (1811.03-9393.31)	118.1 (39.51-214.87)	-5.96 (-6.19 - -5.73)
Democratic Republic of the Congo	5208.42 (1456.18-9745.92)	1296.44 (346.15-2438.05)	67707.99 (18219.24-127242.35)	175.41 (47.19-337.82)	-6.25 (-6.44 - -6.07)
Denmark	300.99 (101.09-503.6)	35.98 (12-60.8)	33.7 (10.44-56.77)	3.71 (1.14-6.41)	-6.82 (-7.55 - -6.08)
Djibouti	2421.02 (790.86-4409.6)	1320.48 (428.21-2422.14)	1349.8 (385.28-2543.8)	324.83 (94.56-628.41)	-4.40 (-4.94 - -3.85)
Dominica	18.95 (5.83-33.67)	76.61 (23.44-137.22)	7.96 (2.31-15.19)	76.86 (22.4-149.86)	0.42 (-0.24 - 1.09)
Dominican Republic	7733.54 (2375.8-14754.92)	272.94 (82.59-518.75)	925.86 (305.56-1837.04)	31.42 (10.06-62.93)	-6.71 (-7.41 - -6.01)
Ecuador	13797.28 (4511.36-23544.76)	358.7 (117.24-611.85)	1830.74 (522.99-3457.78)	38.05 (10.63-73.53)	-7.04 (-7.75 - -6.32)

	[illegible]				
Egypt	519496.17 (164646.83-890807.81)	2150.3 (677.47-3710.72)	79842.2 (25693.45-137134.93)	214.6 (67.68-369.65)	-7.19 (-7.73 - -6.65)
El Salvador	4254.09 (1369.5-7814.33)	193.76 (61.97-358.53)	525.99 (149.72-980.86)	30.17 (8.79-57.44)	-6.10 (-6.74 - -5.47)
Equatorial Guinea	5148.39 (1474.63-9233.56)	2211.89 (631.11-3986.72)	1032.01 (283.19-1992.58)	188.79 (50.68-391.93)	-7.70 (-8.04 - -7.36)
Eritrea	16157.7 (4546.83-29181.72)	917.14 (260.01-1678.12)	5837.96 (1670.42-11100.01)	224.26 (61.94-436.24)	-4.50 (-5.07 - -3.93)
Estonia	581.43 (197.63-942.46)	168.55 (57.38-274.66)	28.81 (9.66-49.17)	14.35 (4.77-24.68)	-7.73 (-8.98 - -6.45)
Eswatini	2404.74 (712.81-4359.75)	582.61 (173.52-1057.22)	783.98 (241.78-1483.92)	194.63 (59.35-370.73)	-3.56 (-4.08 - -3.04)
Ethiopia	238801.76 (66782.38-427422.26)	874.85 (245.06-1568.44)	43585.29 (13282.22-81600.72)	96.05 (29.23-180.41)	-7.06 (-7.42 - -6.70)
Fiji	984.11 (293-1705.07)	362.74 (106.91-638.22)	459.65 (136.47-841.87)	175 (50.92-327.89)	-2.36 (-2.68 - -2.04)
Finland	199.1 (68.49-333.75)	21.85 (7.41-36.69)	26.2 (8.58-44.93)	3.43 (1.09-5.87)	-5.96 (-7.86 - -4.03)
France	2843.85 (972.46-4554.76)	25.33 (8.64-40.77)	518.11 (184.07-857.03)	4.93 (1.69-8.14)	-5.19 (-6.49 - -3.86)
Gabon	2444.77 (691.71-4672.43)	551.34 (155.85-1050.42)	692.83 (201.36-1340.51)	112.45 (32.29-216.06)	-4.98 (-5.23 - -4.74)
Gambia	7996.11 (2571.54-14183.94)	1522.38 (488.02-2715.49)	2170.26 (708.6-3937.1)	213.78 (68.34-394.6)	-6.25 (-7.24 - -5.25)
Georgia	20822.61 (7176.14-34657.6)	1552.77 (535.2-2591.7)	629.94 (214.95-1074.56)	89.71 (30.5-154.26)	-8.84 (-9.90 - -7.76)
Germany	3534.12 (1241.49-5763.96)	27.55 (9.74-45.37)	317.69 (103.8-522.39)	2.71 (0.89-4.53)	-7.12 (-8.07 - -6.17)
Ghana	27037.7 (7575.95-50297.51)	362.23 (100.65-681.36)	9962.47 (2572.62-19526.24)	75.48 (19.25-150.61)	-4.85 (-5.22 - -4.48)
Greece	483.29 (170.79-764.62)	28.68 (10.12-45.64)	101.56 (34.81-169.89)	8.16 (2.72-13.72)	-3.73 (-5.61 - -1.80)
Greenland	36.24 (12.19-60.88)	232.14 (77.34-391.09)	2.76 (0.95-4.85)	23.87 (8.07-43.01)	-6.29 (-6.80 - -5.78)
Grenada	48.52 (15.82-84.88)	141.77 (46.24-250.21)	8.64 (2.47-15.67)	43.14 (12.05-79.22)	-3.37 (-4.89 - -1.82)
Guam	85.45 (29-145.62)	187.95 (63.53-318.28)	37.7 (11.54-67.36)	103.07 (31.38-185.58)	-2.01 (-2.27 - -1.74)
Guatemala	33946.64 (11095.97-58537.16)	784.35 (254.09-1355.29)	11058.16 (3299.41-19919.82)	245.7 (73.47-441.25)	-3.79 (-4.37 - -3.20)
Guinea	89829.86 (25214.27-164693.93)	2728.41 (769.18-5021.83)	30586.56 (8527.8-56837.05)	478.46 (133.42-891.02)	-5.46 (-5.89 - -5.03)
Guinea-Bissau	3495.69 (916.1-6792.95)	655.3 (171.89-1277.82)	1120.61 (287.15-2093.49)	119.31 (31.37-228.05)	-5.32 (-5.84 - -4.80)
Guyana	821.32 (252.04-1379.96)	257.42 (79.19-433.91)	186.73 (54.41-346.92)	87.69 (25.89-164.96)	-3.47 (-4.90 - -2.01)
Haiti	42232.51 (12454.07-78158.27)	1399.97 (413.6-2603.2)	11214.11 (3455.62-20654.97)	250.62 (76.88-468.13)	-5.37 (-5.58 - -5.16)
Honduras	12828.01 (3953.43-22776.98)	548.54 (168.74-980.37)	2332.16 (715-4330.1)	74.16 (22.28-140.19)	-6.28 (-6.59 - -5.96)
Hungary	2167.32 (743.68-3478.23)	120.44 (41.23-193.51)	183.58 (57.81-333.76)	13.97 (4.39-25.43)	-6.47 (-7.66 - -5.26)
Iceland	18.28 (6.51-30.41)	29.85 (10.37-50.59)	3.66 (1.12-6.52)	5.61 (1.68-10.38)	-5.24 (-5.59 - -4.89)
India	5325541.08 (1721622.99-9226394.1)	1603.83 (517.31-2778.84)	986541.91 (298746.12-1744384.27)	307.4 (93.17-547.23)	-5.40 (-5.68 - -5.12)
Indonesia	747139.29 (249701.95-1220579.45)	1167.68 (388.74-1927.56)	130153.73 (43883.25-226256.53)	206.71 (69.35-359.89)	-5.34 (-5.54 - -5.14)
Iran (Islamic Republic of)	79315.2 (24454.16-142604.32)	315.59 (98.31-570.76)	3034.76 (1059.19-5169.11)	15.56 (5.09-27.28)	-9.12 (-9.77 - -8.46)
Iraq	65863.68 (22532.63-114879.79)	740.11 (250.92-1281.75)	13500.4 (4143.36-23488.94)	108.52 (33.6-192.02)	-6.10 (-6.45 - -5.75)
Ireland	407.67 (137.6-665.25)	47.28 (15.93-77.05)	49.52 (15.86-83.5)	5.56 (1.78-9.42)	-6.62 (-7.95 - -5.28)
Israel	498.8 (169.14-802.76)	33.62 (11.33-54.18)	59.61 (18.83-101.79)	2.27 (0.74-3.95)	-8.30 (-9.71 - -6.87)
[illegible]	3053.61 [illegible]	37.98 [illegible]	221.28 [illegible]	[illegible]	-7.60 (-9.15 - [illegible]

Italy	[illegible] (1061.3-4949.96)	[illegible] (13.16-61.34)	[illegible] (71.86-374.76)	3.31 (1.08-5.77)	[illegible] -6.03)
Jamaica	784.84 (247.23-1322.27)	97.88 (30.68-164.43)	104.04 (30.9-198.06)	20.73 (6.07-39.73)	-4.88 (-5.90 - -3.85)
Japan	8566.21 (3058.9-13764.97)	42.57 (14.95-68.72)	802.99 (268.19-1341.34)	5.69 (1.9-9.59)	-6.25 (-7.34 - -5.15)
Jordan	8457.65 (2826.72-13966)	496.04 (166.14-823.13)	2760.72 (931.94-4647.97)	85.02 (29.22-143.38)	-5.54 (-5.88 - -5.21)
Kazakhstan	71637.39 (23730.6-118345.57)	1334.06 (441.93-2205.18)	5596.45 (1933.54-9301.89)	100.87 (34.92-167.85)	-7.87 (-8.79 - -6.94)
Kenya	108334.48 (35517.03-190335.19)	888.62 (291.13-1564.87)	22298.85 (6889.77-40309.49)	129 (38.87-235.23)	-6.24 (-6.56 - -5.92)
Kiribati	438.02 (149.08-729.16)	1312.9 (443.81-2196.66)	161.46 (56.78-283.04)	393.48 (135.56-700.03)	-3.81 (-4.20 - -3.42)
Kuwait	1120.87 (375.64-1853.23)	194.11 (65.25-323.84)	679.51 (228.68-1118.98)	86.94 (29.33-144.69)	-1.17 (-4.06 - 1.80)
Kyrgyzstan	43373.11 (14571.65-70523.51)	2372.07 (793.38-3858.14)	4975.47 (1721.68-8198.56)	218.98 (75.78-362.82)	-7.25 (-8.34 - -6.15)
Lao People's Democratic Republic	101424.48 (32684.77-173543.68)	5019.22 (1615.94-8598.4)	15983.44 (4763.59-29113.33)	676.59 (198.68-1225.33)	-5.83 (-6.06 - -5.60)
Latvia	891.2 (310.09-1429.47)	154.17 (53.29-248.68)	63.36 (21.24-105.63)	23.41 (7.84-39.61)	-5.88 (-7.28 - -4.45)
Lebanon	5029.55 (1418.32-8786.24)	455.3 (129.26-809.99)	947.43 (288.27-1664.29)	79.78 (24.09-141.37)	-5.52 (-5.79 - -5.25)
Lesotho	5636.28 (1761.78-9711.83)	802.88 (248.08-1401.54)	4904.78 (1451.7-8855.28)	834.85 (245.63-1522.28)	0.04 (-0.48 - 0.56)
Liberia	20266.98 (6010.66-38317.69)	1538.31 (453.15-2923.91)	2734.48 (720.37-5121.07)	125.01 (32.69-237.25)	-7.79 (-8.63 - -6.93)
Libya	4634.24 (1547.45-8241.16)	254.89 (83.41-459.75)	996 (320.23-1743.71)	77.73 (22.35-143.52)	-3.43 (-4.76 - -2.08)
Lithuania	875.19 (312.55-1400.9)	105.92 (37.95-171.1)	79.03 (27.43-131.91)	20.71 (7.09-35.06)	-5.24 (-6.60 - -3.85)
Luxembourg	17.02 (5.5-28.36)	25.97 (8.37-43.07)	3.13 (1.06-5.53)	3.25 (1.08-5.86)	-6.72 (-8.32 - -5.09)
Madagascar	148410.27 (48920.55-253807.93)	2440.45 (804.91-4190.73)	43690.17 (13271.74-80775)	373.61 (113.06-689.02)	-5.94 (-6.18 - -5.70)
Malawi	77151.88 (21849.32-142700.25)	1440.05 (406.95-2668.02)	17325.01 (5550.37-32375.18)	221.11 (69.4-420.7)	-5.90 (-6.60 - -5.20)
Malaysia	16491.58 (5187.94-28475.78)	244.3 (76.29-427.28)	3741.51 (1329.99-6204.26)	51.59 (17.38-89.72)	-4.97 (-6.02 - -3.91)
Maldives	933.26 (316.4-1609.33)	794.61 (269.43-1371.07)	72.39 (25.32-121.78)	77.88 (27.3-134.1)	-7.32 (-7.89 - -6.74)
Mali	42636.63 (11577.16-77710.39)	871.25 (236.49-1603.49)	45410.89 (13723.71-84791.6)	350.46 (104.43-659.26)	-2.91 (-3.29 - -2.53)
Malta	32.74 (10.93-54.11)	39.95 (13.25-66.15)	6.24 (1.91-10.83)	9.82 (3.01-17.53)	-4.38 (-5.63 - -3.12)
Marshall Islands	140.06 (44.16-246.18)	656.74 (205.37-1161.58)	59.53 (19.78-104.92)	363.59 (119.77-645.23)	-1.90 (-2.19 - -1.62)
Mauritania	13132.7 (4339.01-22734.65)	1251.09 (412.33-2188.39)	4592.44 (1477.96-8122.45)	244.99 (77.69-438.76)	-5.05 (-5.41 - -4.69)
Mauritius	755.08 (261.1-1226.88)	246.01 (85.32-399.08)	187.8 (61.14-319.98)	100.02 (32.81-171.73)	-2.90 (-4.37 - -1.40)
Mexico	256749.56 (86690.4-423147.85)	762.37 (256.96-1254.8)	12102.86 (3555.61-22507.83)	42.17 (12.36-79.13)	-8.85 (-9.60 - -8.10)
Micronesia (Federated States of)	531.73 (183.4-898.62)	1185.33 (405.93-2007.83)	82.62 (26.28-144.5)	299.22 (93.39-527.36)	-4.34 (-4.71 - -3.97)
Monaco	1.08 (0.33-1.89)	32 (9.55-57.6)	0.51 (0.16-0.9)	10.88 (3.39-19.64)	-3.06 (-3.88 - -2.23)
Mongolia	34998.16 (11760.64-57897.46)	3616.26 (1211.51-5986.61)	3157.43 (1049.39-5498.95)	283.91 (94.34-497.67)	-7.81 (-8.37 - -7.26)
Montenegro	221.93 (73.4-384.88)	146.78 (47.68-259.12)	14.06 (4.59-24.76)	13.15 (4.26-23.98)	-7.27 (-8.12 - -6.41)
Morocco	117558.97 (37059.22-198443.43)	1157.71 (361.64-1965.92)	7259.94 (2355.52-13441.53)	77.55 (24.55-145.59)	-8.37 (-8.49 - -8.24)
Mozambique	107428.5 (30929.1-196466.26)	1570.25 (451.15-2898.25)	31238.02 (8769.94-56717.92)	212.42 (60.46-393.71)	-6.37 (-7.05 - -5.69)
	[illegible]		[illegible]		

Myanmar	609972.75 (200073.47-1050797.46)	4229.15 (1375.58-7325.28)	73611.56 (22565.79-125751.26)	491.16 (149.12-844.65)	-6.75 (-6.89 - -6.61)
Namibia	4807.13 (1618.92-8338.09)	749.6 (249.87-1321.11)	1682.77 (553.91-3124.66)	209.94 (67.79-393.62)	-4.15 (-4.45 - -3.86)
Nauru	46.72 (15.6-79.91)	1009.46 (333.97-1743.78)	24.8 (8.4-43.3)	621.16 (206.76-1095.18)	-1.58 (-1.81 - -1.34)
Nepal	397142.46 (127691-656030.46)	4233.88 (1363.03-7015.39)	26057.27 (8043.66-48921.99)	292.87 (89.88-556.98)	-8.26 (-8.62 - -7.90)
Netherlands	844.15 (290.93-1349.46)	31.43 (10.73-50.36)	112.47 (35.93-194.06)	4.44 (1.42-7.74)	-5.84 (-6.92 - -4.74)
New Zealand	547.41 (190.55-906.26)	68.51 (23.86-113.74)	73.21 (23.55-126.8)	8.12 (2.59-14.16)	-7.14 (-9.04 - -5.19)
Nicaragua	13959.76 (4456.53-24443.26)	737.58 (235.47-1293.42)	1496.85 (448.18-2716.02)	80.16 (24.12-146.52)	-7.01 (-7.62 - -6.41)
Niger	120592.2 (35536.3-222796.3)	2516.28 (734.27-4675.62)	49709.73 (13757.13-91298.08)	344.66 (94.94-635.79)	-6.20 (-6.77 - -5.61)
Nigeria	513252.81 (150731.07-918296.09)	1138.8 (332.48-2042.14)	252065.46 (71525.8-471651.1)	238.42 (67.29-447.5)	-4.95 (-5.21 - -4.69)
Niue	3.29 (1.01-5.78)	444.17 (137.32-791.78)	3.11 (1.08-5.22)	904.67 (312.36-1541.62)	2.70 (1.23 - 4.20)
North Macedonia	2910.5 (926.03-4987.94)	596.22 (189.35-1024.94)	70.87 (23.55-123.07)	23.8 (7.86-42.2)	-9.83 (-10.95 - -8.70)
Northern Mariana Islands	22.95 (8.07-40.65)	171.6 (59.61-307.76)	5.66 (1.92-9.78)	58.8 (19.74-102.72)	-3.46 (-4.59 - -2.31)
Norway	199.06 (72.71-325.88)	25.24 (9.21-41.31)	15.84 (5.24-27.56)	1.88 (0.63-3.32)	-8.40 (-10.09 - -6.68)
Oman	2691.92 (856.82-4953.04)	290.83 (92.25-541.99)	459.32 (144.94-820.41)	37.86 (11.59-68.59)	-6.61 (-7.38 - -5.83)
Pakistan	884268.73 (286328.44-1466165.2)	1679.92 (543.4-2786.26)	355044.15 (115116.78-631651.16)	417.81 (136.23-744.33)	-4.44 (-4.88 - -4.01)
Palau	23.03 (7.55-41.31)	542.47 (176.88-974.2)	5.1 (1.6-8.58)	185.15 (56.95-313.72)	-3.37 (-3.62 - -3.12)
Palestine	5103.46 (1733.08-8943.87)	465.44 (157.26-821.45)	1271.63 (418.92-2170.81)	71.12 (22.69-123.51)	-5.84 (-6.22 - -5.46)
Panama	1194.92 (375.57-2083.47)	146.11 (46.01-255.82)	435.91 (131.43-794.37)	40.75 (12.33-74.23)	-4.21 (-5.20 - -3.20)
Papua New Guinea	57664.62 (19321.14-96811.87)	3121.53 (1044.03-5255.07)	67504.94 (23522.14-118541.02)	1557.3 (535.2-2757.41)	-2.24 (-2.51 - -1.98)
Paraguay	5740.78 (1857.4-10112.52)	327.14 (105.37-576.38)	765.72 (218.97-1520.69)	40.43 (11.08-82.05)	-6.72 (-7.85 - -5.58)
Peru	68487.93 (21555.16-120671.12)	819.73 (257.12-1445.78)	5024.74 (1450.42-9479.35)	53.15 (15.15-101.88)	-8.69 (-9.80 - -7.58)
Philippines	396005.21 (129369.02-668391.64)	1514.55 (497.83-2549.75)	82147.39 (27190.05-141279.1)	253.13 (82.33-442.14)	-5.74 (-6.20 - -5.28)
Poland	9919.95 (3507.78-15642.98)	116.81 (40.72-184.32)	951.41 (313.13-1600.05)	17.03 (5.61-28.97)	-5.88 (-7.88 - -3.83)
Portugal	1586.26 (548.1-2640.34)	91.32 (31.33-152.19)	100.35 (32.2-166.07)	7.83 (2.53-13.33)	-7.66 (-9.76 - -5.52)
Puerto Rico	349.47 (107.8-599.87)	37.98 (11.65-65.45)	23.96 (7.28-43.12)	7.71 (2.32-13.93)	-4.75 (-5.48 - -4.02)
Qatar	152.57 (45.54-283.99)	107.71 (31.64-200.54)	70.69 (23.63-122.89)	13.68 (4.39-24.26)	-6.46 (-6.82 - -6.10)
Republic of Korea	11625.54 (3995.18-19667.74)	114.74 (39.24-196.47)	213 (69.84-366.75)	4.04 (1.27-7.24)	-10.18 (-10.70 - -9.66)
Republic of Moldova	7741.71 (2672.44-12888.7)	628.71 (216.45-1046.56)	503.28 (173.51-871.5)	111.84 (38.25-193.71)	-5.30 (-6.40 - -4.18)
Romania	59341.9 (20429.37-96840.16)	1168.2 (401.6-1908.72)	3130.27 (1069.28-5204)	114.53 (38.46-189.65)	-6.93 (-8.04 - -5.80)
Russian Federation	111630.62 (39867.49-178546.38)	335.05 (119.49-536.24)	9290.5 (3251.14-15426.81)	41.03 (14.09-68.25)	-6.45 (-7.41 - -5.48)
Rwanda	53876.67 (15350.94-98570.27)	1414.25 (397.8-2621.22)	7433.54 (2034.98-13774.58)	148.98 (40.2-282.51)	-6.98 (-8.11 - -5.83)
Saint Kitts and Nevis	13.81 (4.46-23.92)	102.57 (32.83-178.16)	3.92 (1.23-7.34)	44.09 (13.52-83.75)	-2.56 (-3.52 - -1.58)
Saint Lucia	38.67 (12.12-68.86)	76.5 (24.05-136.33)	7.2 (2.07-13.36)	27.71 (7.87-53.01)	-3.17 (-3.92 - -2.40)
Saint Vincent and [illegible]	[illegible]	114.1 [illegible]	[illegible]	34.56 [illegible]	-3.64 (-4.41 - [illegible]

Saint Vincent and the Grenadines	42.2 (13.71-76.57)	[illegible] (36.85-207.47)	7.49 (2.29-13.65)	[illegible] (10.18-64.23)	[illegible] -2.88)
Samoa	493.12 (157.83-865.55)	678.72 (214.74-1192.4)	186.6 (55.35-349.02)	224.62 (65.86-421.8)	-3.49 (-3.79 - -3.20)
San Marino	0.98 (0.32-1.74)	28.09 (9.01-50.81)	0.07 (0.02-0.13)	1.92 (0.57-3.71)	-8.18 (-8.50 - -7.87)
Sao Tome and Principe	196.99 (53.91-367.31)	338.02 (92.08-629.46)	19.5 (5.38-38.92)	26.58 (7.2-54.01)	-7.77 (-8.75 - -6.78)
Saudi Arabia	20974.3 (6269.36-38025.3)	305.34 (92.07-560.33)	1110.9 (370.1-2073.53)	15.35 (4.88-29.99)	-9.28 (-9.51 - -9.04)
Senegal	69706.42 (21546-119769.47)	1679.29 (516.62-2894.51)	13422.58 (4032.8-24613.98)	207.4 (62.63-386.26)	-6.48 (-7.43 - -5.51)
Serbia	5327.02 (1850.51-9578.2)	271.18 (92.52-491.58)	184.09 (62.45-305.75)	16.55 (5.68-28.4)	-7.86 (-10.36 - -5.30)
Seychelles	65.02 (21.5-108.72)	280.88 (94.03-476.88)	35.58 (11.06-61.89)	157.74 (48.3-276.79)	-1.76 (-2.69 - -0.83)
Sierra Leone	77492.45 (22942.05-139425.78)	3535.57 (1044.31-6354.01)	16221.62 (4767-30757.84)	427.74 (125.31-828.45)	-6.60 (-7.14 - -6.06)
Singapore	846.95 (279.55-1446.13)	140.91 (46.81-243.83)	91.63 (29.24-159.61)	11.34 (3.58-19.92)	-7.77 (-9.47 - -6.04)
Slovakia	3737.78 (1278.76-6075.39)	314.19 (106.46-516.01)	484.87 (160.18-836.11)	58.88 (19.55-102.78)	-5.33 (-6.62 - -4.02)
Slovenia	510.43 (181.37-836.06)	141.45 (49.92-232.94)	69.29 (23.56-117.07)	24.08 (8.25-41.03)	-5.46 (-6.60 - -4.32)
Solomon Islands	2680.23 (893.92-4466.54)	1582.48 (530.58-2654.49)	1647.74 (536.72-2836.62)	608.45 (194.23-1055.07)	-3.07 (-3.63 - -2.51)
Somalia	72131.8 (19215.02-132685.05)	1645.31 (437.18-3048.35)	51298.67 (13750.11-97901.04)	441.1 (118.05-846.78)	-4.08 (-4.53 - -3.63)
South Africa	191814.36 (62702.15-327164.4)	1379 (450.72-2355.99)	37709.84 (12733.73-65094.09)	262.42 (88.8-459.64)	-5.42 (-5.74 - -5.11)
South Sudan	48691.73 (14968.94-90305.26)	1682.89 (516.52-3131.95)	27664.35 (8488.1-52721.01)	622.93 (191.69-1197.18)	-3.25 (-3.75 - -2.74)
Spain	2284.71 (795.04-3602.4)	35.91 (12.48-56.99)	223.34 (77.53-372.71)	3.86 (1.3-6.47)	-6.97 (-8.47 - -5.44)
Sri Lanka	8904.48 (3119.22-14930.58)	171.43 (59.16-288)	1123.3 (305.58-2116.53)	23.9 (6.52-45.92)	-6.13 (-7.43 - -4.81)
Sudan	152748.79 (46165.02-284848.67)	1542.45 (461.33-2886.49)	19688.8 (5724.62-36263.75)	121.59 (35.39-227.45)	-7.86 (-8.06 - -7.65)
Suriname	464.08 (160.7-798.67)	369.63 (127.21-638.41)	119.98 (35.72-224.68)	92.94 (27.62-175.8)	-4.36 (-5.09 - -3.63)
Sweden	248.59 (82.39-413.25)	15.57 (5.13-26.22)	25.26 (7.93-43.73)	1.47 (0.46-2.63)	-7.54 (-8.87 - -6.19)
Switzerland	361.27 (129.08-594.61)	31.68 (11.28-52.18)	41.59 (13.64-70.19)	3.24 (1.04-5.5)	-7.08 (-8.74 - -5.38)
Syrian Arab Republic	35213.68 (10992.36-63115.64)	574.72 (176.23-1042.53)	2574.99 (751.37-4608.39)	79.35 (22.34-146.71)	-6.31 (-7.25 - -5.37)
Taiwan (Province of China)	7174.1 (2414.15-11687.54)	150.84 (50.87-246.49)	792.08 (264.8-1367.13)	30.22 (10.06-52.17)	-5.05 (-6.57 - -3.52)
Tajikistan	73168.09 (24392.96-126716.38)	2728.87 (910.6-4740.04)	18488.3 (5385.44-33448.23)	486.26 (140.86-888.55)	-5.28 (-5.50 - -5.05)
Thailand	58560.57 (20480.06-97866.68)	388.31 (129.09-665.27)	6148.88 (2044.79-10468.9)	72.34 (23.77-124.72)	-5.27 (-5.67 - -4.86)
Timor-Leste	19161.37 (6483.71-32859.77)	4826.51 (1636.84-8294)	4142.95 (1442.48-7190.41)	785.88 (272.83-1371.47)	-5.61 (-5.94 - -5.28)
Togo	16755.43 (5071.02-29742.02)	860.85 (259-1542.81)	5561.7 (1456.15-10463.09)	166.25 (43.2-314.11)	-5.23 (-5.52 - -4.95)
Tokelau	3.42 (1.03-5.98)	608.78 (184.29-1075.71)	3.31 (1.2-5.58)	1113.55 (395.99-1893.85)	3.11 (1.29 - 4.96)
Tonga	249.35 (83.72-423.68)	573.39 (190.82-983.3)	93.68 (29.9-168.47)	229.14 (71.84-412.87)	-2.88 (-3.28 - -2.48)
Trinidad and Tobago	525.9 (164.24-875)	136.64 (42.43-227.39)	91.37 (27.81-164.82)	38.65 (11.55-69.87)	-3.99 (-4.55 - -3.42)
Tunisia	24288.42 (7669.12-49334.05)	795.59 (250.57-1631.73)	1579.59 (544.17-3019.64)	60.95 (20.07-121.17)	-7.97 (-8.33 - -7.61)
Turkey	300648.62 (106546.32-526442.87)	1510.92 (532.87-2655.83)	8711.62 (2875.67-15040.52)	52.21 (17.31-91.94)	-10.27 (-10.63 - -9.91)
[illegible]	50278.9 [illegible]	[illegible]	8665.42 [illegible]	[illegible]	[illegible]

Turkmenistan	[illegible] (16449.37-83949.62)	3018.26 (989.11-5038.67)	[illegible] (2847.32-14903.21)	[illegible] (184.59-976.21)	[illegible] -4.42)
Tuvalu	110.33 (36.11-193.82)	2585.86 (842.89-4560.06)	13.62 (4.6-23.43)	370.82 (123.87-643.3)	-6.07 (-6.32 - -5.82)
Uganda	65683.77 (18957.73-122444.28)	650.02 (185.82-1237.7)	25604.57 (6730.9-48891.75)	124 (31.62-238.62)	-5.25 (-5.88 - -4.62)
Ukraine	23891.34 (8635.64-39202.43)	220.95 (79.51-363.95)	2009.28 (670.47-3338.25)	41.15 (13.84-69.54)	-5.06 (-6.64 - -3.46)
United Arab Emirates	1015.93 (350.23-1780.27)	158.63 (54.29-283.96)	234.88 (82.24-400.44)	18.55 (6.15-33.14)	-6.55 (-7.28 - -5.81)
United Kingdom	4251.22 (1504.57-7016.61)	2370.01 (753.71-4168.35)	633.65 (198.3-1086.7)	242.62 (69.59-453.26)	-5.93 (-7.59 - -4.24)
United Republic of Tanzania	323054.52 (102696.48-566925.45)	2370.01 (753.71-4168.35)	60991.08 (17768.46-113710.55)	242.62 (69.59-453.26)	-7.19 (-7.50 - -6.88)
United States of America	18019.93 (6139.73-29674.93)	31.99 (10.92-52.76)	3123.76 (1003.8-5378.5)	5.66 (1.78-9.8)	-5.35 (-6.22 - -4.48)
United States Virgin Islands	13.02 (3.85-23.55)	41.29 (11.84-76.1)	0.7 (0.21-1.33)	6.11 (1.82-11.8)	-5.95 (-6.25 - -5.65)
Uruguay	1353.28 (475.24-2191.29)	173.19 (60.82-281.7)	150.87 (47.83-256.64)	26.14 (8.03-45.57)	-5.55 (-6.43 - -4.67)
Uzbekistan	116398.62 (36890.18-198983.04)	1214.56 (385.07-2079.25)	45740.65 (15107.77-77566.75)	421.06 (137.27-720.15)	-3.18 (-3.61 - -2.75)
Vanuatu	476.03 (145.96-876.87)	622.81 (191.76-1160.07)	264.61 (78.9-470.63)	220.58 (64.32-398.41)	-3.11 (-3.95 - -2.27)
Venezuela (Bolivarian Republic of)	21230.92 (7274.91-35088.78)	294.21 (100.94-487.26)	5319.38 (1679.6-9636.85)	84.34 (26.6-153.32)	-3.71 (-5.17 - -2.24)
Viet Nam	268177.2 (100824.4-446749.4)	997.78 (373.44-1665.58)	31754.99 (9984.78-55482.14)	135.77 (42.01-238.8)	-6.22 (-6.50 - -5.94)
Yemen	131225.32 (41340.26-239480.82)	1660.76 (519.04-3022.03)	31302.67 (10726.11-55935.61)	232.4 (75.89-418.6)	-6.14 (-6.48 - -5.80)
Zambia	83720.92 (25771.19-150765.97)	1955.29 (597.41-3523.35)	20912.74 (6011.32-37998.17)	250.62 (72.81-457.95)	-6.45 (-6.73 - -6.16)
Zimbabwe	35244.08 (10627.73-63614.8)	700.86 (210.75-1270.96)	30536.71 (9325.08-53628.76)	483.81 (147.63-850.39)	-1.04 (-1.64 - -0.44)

DALYs, Disability-Adjusted Life Years; ASRs, Age-Standardized Rates; AAPC, Average Annual Percentage Change

**Supplementary Table S3.Global Forecast of ASMR and ASDR in Children Under 14 from 2022 to 2035**		
Year	ASMR	ASDR
2022	2.03	181.33
2023	1.8	161.14
2024	1.59	143.15
2025	1.41	127.12
2026	1.25	112.87
2027	1.1	100.22
2028	0.98	88.97
2029	0.86	78.97
2030	0.76	70.07
2031	0.68	62.18
2032	0.6	55.18
2033	0.53	48.96
2034	0.47	43.45
2035	0.41	38.56

ASMR, Age-Standardized Mortality Rates; ASDR, Age-Standardized Disability-adjusted life year Rates

© **2026 Yang F. et al.**
